# Chemical Constituents of the Mushroom *Dictyophora indusiata* and Their Anti-Inflammatory Activities

**DOI:** 10.3390/molecules28062760

**Published:** 2023-03-18

**Authors:** Yingfang Zhang, Hang Xun, Quan Gao, Feifei Qi, Jia Sun, Feng Tang

**Affiliations:** 1 Key Laboratory of National Forestry and Grassland Administration Beijing for Bamboo & Rattan Science and Technology, International Centre for Bamboo and Rattan, Beijing 100102, China; zhangyingfang@ipe.ac.cn (Y.Z.); xunhang@icbr.ac.cn (H.X.); 2 Anhui Key Laboratory of Agricultural Products, School of Resource and Environment, Anhui Agricultural University, Hefei 230036, China; 15155139755@163.com; 3 Shandong Provincial Key Laboratory of Synthetic Biology, Qingdao Institute of Bioenergy and Bioprocess Technology, Chinese Academy of Sciences, Qingdao 266101, China; qiff@qibebt.ac.cn; 4 Eurofins Agroscience Services, Hercules, CA 94547, USA

**Keywords:** *Dictyophora indusiata*, structure elucidation, sesquiterpenoids, ergosterol derivatives, anti-inflammatory activity, molecular docking

## Abstract

As an edible and medicinal fungus, *Dictyophora indusiata* is well-known for its morphological elegance, distinctive taste, high nutritional value, and therapeutic properties. In this study, eighteen compounds (**1**–**18**) were isolated and identified from the ethanolic extract of *D. indusiata*; four (**1**–**4**) were previously undescribed. Their molecular structures and absolute configurations were determined via a comprehensive analysis of spectroscopic data (1D/2D NMR, HRESIMS, ECD, and XRD). Seven isolated compounds were examined for their anti-inflammatory activities using an in vitro model of lipopolysaccharide (LPS)-simulated BV-2 microglial cells. Compound **3** displayed the strongest inhibitory effect on tumor necrosis factor-α (TNF-α) expression, with an IC_50_ value of 11.9 μM. Compound **16** exhibited the highest inhibitory activity on interleukin-6 (IL-6) production, with an IC_50_ value of 13.53 μM. Compound **17** showed the most potent anti-inflammatory capacity by inhibiting the LPS-induced generation of nitric oxide (NO) (IC_50_: 10.86 μM) and interleukin-1β (IL-1β) (IC_50_: 23.9 μM) and by significantly suppressing induced nitric oxide synthase (iNOS) and phosphorylated nuclear factor-kappa B inhibitor-α (*p*-IκB-α) expression at concentrations of 5 μM and 20 μM, respectively (*p* < 0.01). The modes of interactions between the isolated compounds and the target inflammation-related proteins were investigated in a preliminary molecular docking study. These results provided insight into the chemodiversity and potential anti-inflammatory activities of metabolites with small molecular weights in the mushroom *D. indusiata*.

## 1. Introduction

*Dictyophora indusiata*, belonging to the family *Phallaceae* (phylum Basidiomycetes) of the Agaricomycetes class of fungi, is famous for its beautiful morphological features. Due to its delicious taste, distinctive fragrance, and medicinal properties, *D. indusiata* has a long history as a healthy food and as a component of folk medicine [1]. Traditional Chinese medicine has widely recognized that *D. indusiata* benefits some chronic diseases by alleviating inflammatory symptoms [2,3]. It is reasonable to suppose the existence of metabolites in *D. indusiata* that offer therapeutic potential.

Most studies on the molecular basis of the nutritional and medicinal values of *D. indusiata* have focused on fungal biomacromolecules, such as carbohydrates and proteins. Polysaccharides are found in the highest concentrations in the mature fruiting body of *D. indusiata* and, therefore, are the most intensively studied chemical constituents of *D. indusiata* [4]. It has been proven that *D. indusiata* polysaccharides (DIPs) have effective anti-tumor, anti-oxidative, anti-inflammatory, immune-enhancing, and anti-diabetes properties [5]. Compared to the polysaccharides, the bioactive compounds with small molecular weights in *D. indusiata* have not been sufficiently investigated [1]. From this mushroom species, three eudesmane-type sesquiterpenes and five monoterpene alcohols were reported to be identified by Kawagishi et al. [6,7]; albaflavenone and two derivatives (9,10-dihydroxy-albaflavenone and 5-hydroxy-albaflavenone) were isolated by Huang et al. and Zhang et al., respectively [8,9]; two linear sesquiterpene carboxylic acids (Phallac acids A and B) were obtained and determined by Lee et al. [10]. In addition to the terpenoid compounds, Lee et al. identified three alkaloids, Dictyoquinazols A, B, and C, from *D. indusiata* [11]; and Sharma et al. reported the isolation of 5-hydroxymethyl–2-furfural (HMF) [12]. The biological investigations revealed that some of these small molecules isolated from *D. indusiata* possess neuroprotective, anti-tyrosinase, anti-glucosidase, and some anti-inflammatory activities [6,9,10,11,12]. However, the role of small-molecular-weight metabolites in the anti-inflammatory property of the mushroom *D. indusiata* has yet to be fully elucidated.

Inflammation is a natural response of the immune system to damage from physical, chemical, and pathogenic factors. The activation of the nuclear factor kappa-light-chain-enhancer of activated B cells (NF-κB) pathway plays a central role in acute and chronic inflammation [13]. Abnormally high NF-κB activity is highly associated with aberrant expressions of a series of inflammatory cytokines and other mediators, and it is further associated with many diseases, such as cancer, diabetes, Alzheimer’s disease, and cardiac diseases. It has been revealed that the efficient strategy for alleviating inflammatory symptoms involves either reducing the expression of inflammatory cytokines via mediation of the related signaling pathways or by inhibiting their actions using antibodies. In the clinical therapies used to cure inflammatory disease, several cytokine inhibitors, such as Etanercept, Adalimumab, and Infliximab, have been successfully applied. However, the high costs and the side effects of adverse immunological reactions limit their applications [14]. Hence, the discovery of safe and efficacious anti-inflammatory agents is still in urgent demand.

Edible and medicinal fungi are currently seen as important sources of active pharmaceutical ingredients, which can serve as lead molecules in the development of novel drugs. In this context, numerous types of bioactive natural products have been isolated from various fungi [15,16,17]. In the literature, it is abundantly clear that the anti-inflammatory effect of *D. indusiata* polysaccharides has long been recognized [5,18,19,20]. However, the potential anti-inflammatory capacity and underlying mechanism of other ingredients in *D. indusiata* are still unclear. Herein, we report on the systematical isolation and identification of the chemical constituents in the ethanolic extract of *D. indusiata*. The inhibitory effects of the isolated compounds against lipopolysaccharide (LPS)-induced inflammatory responses in mouse BV-2 microglial cells were evaluated, and their anti-inflammatory mechanisms of action were investigated using molecular docking simulations. The results of this study might contribute to the knowledge of the chemistry of *D. indusiata* and the discovery of fungal natural products possessing potent anti-inflammatory efficiency with reliable safety profiles.

## 2. Results

### 2.1. Structure Elucidation of the Isolated Compounds

**Figure 1:**
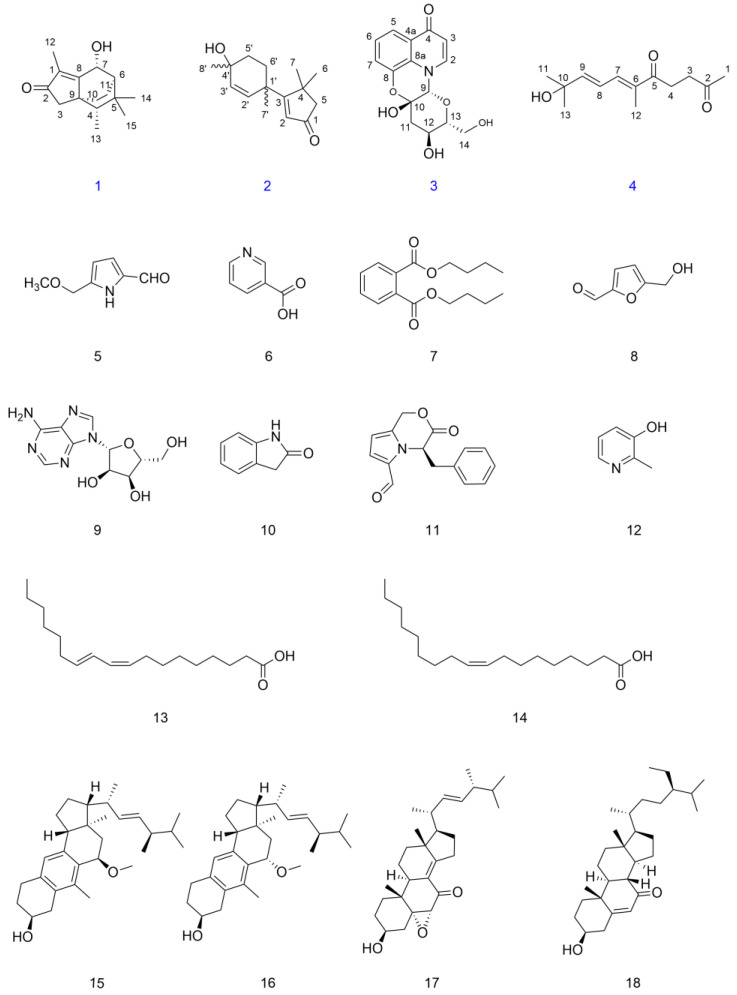
Structures of compounds **1**–**18** isolated from *Dictyophora indusiata*. The blue color indicates the newly identified compounds.

Eighteen compounds were isolated from the ethyl acetate partitioned *Dictyophora indusiata* ethanolic extract. On the basis of the comprehensive analysis of spectroscopic data from high resolution electrospray ionization mass spectra (HRESIMS), 1D/2D nuclear magnetic resonance (NMR) spectra, ultraviolet (UV) spectra, and comparison with the references, four previously undescribed compounds were named Indusiataines A–D (**1**–**4**). Fourteen known compounds were elucidated to be 5-(Methoxymethyl)-1*H*-pyrrole-2-carbaldehyde (**5**) [21], nicotinic acid (**6**) [22], dibutyl phthalate (**7**) [23], 5-hydroxymethyl-2-furancarboxaldehyde (**8**) [24], adenosine (**9**) [25], oxindole (**10**) [26], 3-oxo-4-benzyl-3,4-dihydro-1*H*-pyrrolo [2,1-c] oxazine-6-methylal (**11**) [27], 2-methyl-3-pyridinol (**12**) [28], linoleic acid (**13**) [29], oleic acid (**14**) [30], 1(10→6)abeo-ergosta-5,7,9,22-tetraen-11β-methoxy-3α-ol (**15**) [31], citreoanthrasteroid (**16**) [31], 5α,6α-epoxy-3β-hydroxy-(22*E*)-ergosta-8(14),22-dien-7-one (**17**) [32], and 7-ketositosterol (**18**) [33] (Figure 1). The UV, HRESIMS, and NMR spectra of compounds **1**–**4** are provided in Figure S1.

**Figure 2:**
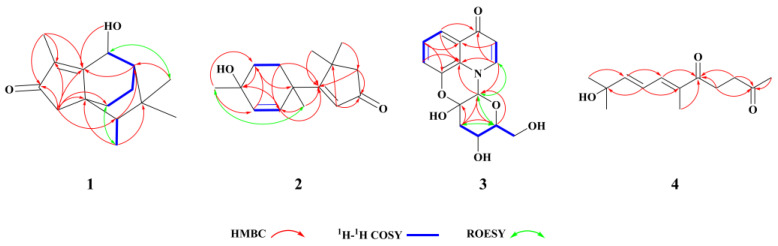
Key HMBC, COSY, and ROESY correlations of compounds **1**–**4**.

**Figure 3:**
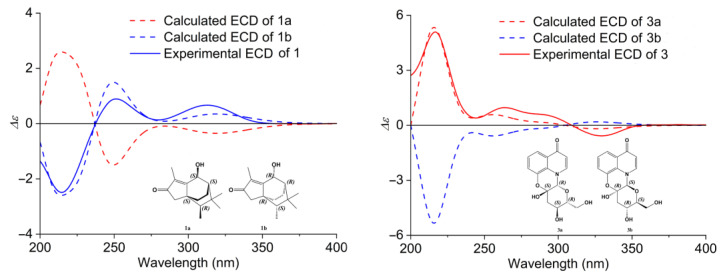
The experimental ECD spectra and calculated ECD curves of compounds **1** and **3**.

**Figure 4:**
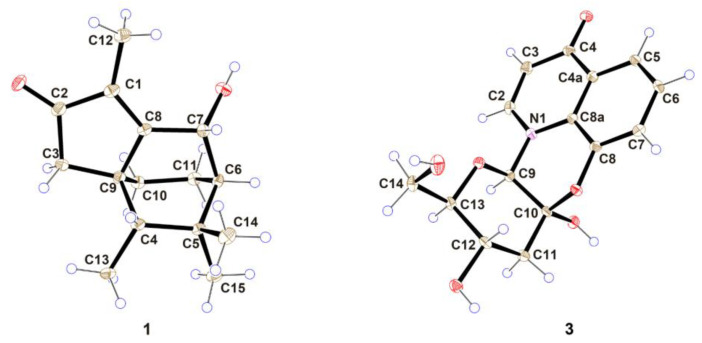
ORTEP (Oak Ridge Thermal-Ellipsoid Plot Program) plots of the X-ray crystal structure of compounds **1** and **3**. Ellipsoids are drawn at the 35% probability level.

Compound **1** was obtained as a yellowish amorphous powder. Its molecular formula, C_15_H_22_O_2_, was established by positive HRESIMS (*m/z* 235.1690 [M + H]^+^, calculated 235.1693), with an index of hydrogen deficiency (IHDs) of five. The UV spectrum of **1** exhibited an absorption maximum of 248.7 nm (Figure S1). The NMR data are provided in Table 1, chemical shifts (*δ*) are reported in parts per million (ppm), and coupling constants (*J*) are given in Hz. The ^1^H NMR data of compound **1** demonstrated three methine hydrogen signals at *δ*_H_ 4.82 (1H, *s*), *δ*_H_ 1.36 (1H, *m*), and *δ*_H_ 1.14 (1H, *m*); three methylenes at *δ*_Ha_ 2.08 (1H, *d*, *J* = 18.0) and *δ*_Hb_ 1.86 (1H, *d*, *J* = 18.0), *δ*_Ha_ 1.91 (1H, *m*) and *δ*_Hb_ 0.98 (1H, *m*), and *δ*_Ha_ 1.78 (1H, *m*) and *δ*_Hb_ 1.58 (1H, *m*); and four methyls at *δ*_H_ 1.69 (3H, *s*), *δ*_H_ 0.94 (6H, *d*, *J* = 1.5), and *δ*_H_ 0.86 (3H, *d*, *J* = 7). The ^13^C-NMR spectrum and DEPT spectrum revealed the resonances of 15 carbons (Table 1), which included one ketone carbon (*δ*_C_ 208.4), two olefinic carbons (*δ*_C_ 181.5 and 132.9), two quaternary carbons (*δ*_C_ 45.2 and 34.0), three methines (*δ*_C_ 65.1, 46.8, and 43.6), three methylenes (*δ*_C_ 44.7, 25.4, and 16.6), and four methyls (*δ*_C_ 30.3, 25.2, 11.6, and 8.5). The other eleven carbons and the unassigned three IHDs suggested that compound **1** possessed a tricyclic core group. A cyclopentenone ring (ring A, which contained C-1, C-2, C-3, C-8, and C-9) was furnished by the HMBC correlations (Figure 2) of H-3 (*δ*_H_ 2.08 and 1.86) to C-1 (*δ*_C_ 132.9), C-2 (*δ*_C_ 208.4), C-8 (*δ*_C_ 181.5), and C-9 (*δ*_C_ 45.2), and from H-12 (*δ*_H_ 1.69) to C-1, C-2 and C-8. The ^1^H-^1^H COSY correlations of H-10 (*δ*_H_ 1.91, 0.98)/H-11 (*δ*_H_ 1.78, 1.58), H-11/H-6 (*δ*_H_ 1.36), and H-6/H-7 (*δ*_H_ 4.82) suggested that the connections were C-10 (*δ*_C_ 25.4) to C-11 (*δ*_C_ 16.6), C-11 to C-6 (*δ*_C_ 46.8), and C-6 to C-7 (*δ*_C_ 65.1). In combination with HMBC correlations from H-6 (*δ*_H_ 1.36) to C-8, H-3 to C-10 (*δ*_C_ 25.4), and 7-OH (*δ*_H_ 5.19) to C-8 indicated that C-8 and C-9 were involved in further cyclization, leading to a cyclohexane ring (ring B) fused to ring A at C-8 and C-9; ring B was composed of C-6, C-7, C-8, C-9, C-10, and C-11. HMBC correlations of H-13 (*δ*_H_ 0.86) with C-5 (*δ*_C_ 34.0), C-9 (*δ*_C_ 45.2), and H-14,15 (*δ*_H_ 0.94) with C-6 suggested that C-6 and C-9 were the bridgehead carbons of another cyclohexane ring (ring C), which contained C-4, C-5, C-6, C-7, C-8, and C-9. Furthermore, four methyls at H-12, H-13, H-14, and H-15, along with HMBC correlations of H-12/C-1, H-13/C-4, and H-14, 15/C-5, confirmed the connection of the methyl group. Compound **1** was finally established, as depicted in Figure 1, with a fused tricyclic system comprised of a cyclopentenone (ring A) and two cyclohexane (rings B and C) fragments. The relative configuration was further deduced from ROESY data (Figure 2), and key correlations between H-10 and H-13 and between H-7 and H-14 or -15 suggested that H-10, H-11, H-13, and 7-OH were on the same face of ring B. Thus, the relative configuration for compound **1** could be considered a pair of enantiomers of 4*R*′,6*S*′,7*S*′,9*S*′. TD-DFT ECD calculations confirmed the absolute configuration of compound **1**, and the structure was optimized by functional analysis of ωB97XD with the TZVP basis set and MeOH as SMD. The obtained calculated ECD spectra (Figure 3) of (4*S*,6*R*,7*R*,9*R*)-9b showed a complete match to the measured spectrum. The absolute configuration of compound **1** was further confirmed by single-crystal X-ray diffraction with a suitable Flack parameter of 0.04 (Figure 4). The absolute configuration of compound **1** was determined to be 4*S*,6*R*,7*R*,9*R*, and it was named Indusiataine A.

Compound **2** was purified as a yellowish oil. Its molecular formula, C_15_H_22_O_2_, was established by positive HRESIMS (*m/z* 235.1694 [M + H]^+^, calculated 235.1693), with an index of hydrogen deficiency (IHDs) of five. The UV spectrum exhibited an absorption maximum of 235.7 nm (Figure S1). The ^1^H NMR spectrum (Table 1) displayed signals for two double bonds, which included three hydrogen signals at *δ*_H_ 5.88 (1H, *s*), *δ*_H_ 5.72 (1H, *dd*, *J* = 10.0, 1.0), and *δ*_H_ 5.56 (1H, *dd*, *J* = 10.0, 1.0); three methylenes at *δ*_H_ 2.25 (2H, *d*, *J* = 11.0), *δ*_Ha_ 1.92 (1H, *m*) and *δ*_Hb_ 1.74 (1H, *m*) and at *δ*_Ha_ 1.61 (1H, *m*) and *δ*_Hb_ 1.40 (1H, *m*); and four methyls at *δ*_H_ 1.34 (3H, *s*), *δ*_H_ 1.28 (3H, *s*), *δ*_H_ 1.26 (3H, *s*), and *δ*_H_ 1.10 (3H, *s*) in the ^1^H NMR spectrum as well as active hydrogen signals at *δ*_H_ 4.55 (1H, *s*). The ^13^C-NMR and DEPT spectra (Table 1) showed carbon signals corresponding to the chemical units described above and confirmed their presence. These resonances included one ketone carbon (*δ*_C_ 206.8), three olefinic carbons (*δ*_C_ 135.1, *δ*_C_ 132.9, and *δ*_C_ 129.3), and three odd numbers, which indicated that *δ*_C_ 193.5 prefers one olefinic carbon, not to a ketone carbon; three quaternary carbons (*δ*_C_ 66.2, *δ*_C_ 44.9, and *δ*_C_ 41.4), three methines (*δ*_C_ 54.8, *δ*_C_ 35.3 and *δ*_C_ 34.3), and four methyl carbons (*δ*_C_ 29.9, *δ*_C_ 29.5, *δ*_C_ 29.4, and *δ*_C_ 28.5). In the HMBC spectrum (Figure 2), correlations of H-2 (*δ*_H_ 5.88) to C-4 (*δ*_C_ 44.9), C-5 (*δ*_C_ 54.8), and C-1 (*δ*_C_ 206.8) and from H-5 (*δ*_H_ 2.25) to C-1 (*δ*_C_ 206.8) and C-3 (*δ*_C_ 193.5), suggesting that a cyclopentenone (ring A) was formed by C-1, C-2 (*δ*_C_ 129.3), C-3, C-4, and C-5; a cyclohexene ring (ring B) formed by C-1′ (*δ*_C_ 41.4), C-2′ (*δ*_C_ 132.9), C-3′ (*δ*_C_ 135.1), C-4′ (*δ*_C_ 66.2), C-5′ (*δ*_C_ 35.3), and C-6′ (*δ*_C_ 34.3) was readily furnished by the ^1^H-^1^H COSY correlations (Figure 2) of H-2′ (*δ*_H_ 5.72)/H-3′ (*δ*_H_ 5.56) and H-5′ (*δ*_H_ 1.61, 1.40)/H-6′ (*δ*_H_ 1.92, 1.72), in combination with HMBC correlations of H-2′, H-3′, H-5′ (*δ*_H_ 1.61, 1.40) and H-6′ (*δ*_H_ 1.92, 1.74) to C-1′ and C-4′. HMBC correlations of H-6 (*δ*_H_ 1.34) and H-7 (*δ*_H_ 1.26) with C-3 and C-5, H-7′ (*δ*_H_ 1.28) with C-3 and C-2′, and H-8′ (*δ*_H_ 1.10) with C-3′ and C-5′, which indicated four methyl positions, as depicted in Figure 2, respectively. The relative configuration was further deduced from ROESY data (Figure 2). Key correlations between H-7′ and H-8′ suggested that H-7′ and H-8′ were on the same face of ring B. Therefore, a pair of enantiomers of 1′*S*′, 4′*R*′ was possible for the relative configuration of compound **2**. The relative configuration of compound **2** was established as 1′*S*, 4′*R*, and named Indusiataine B.

Compound **3** was isolated as a brown oil, and after recrystallization in methanol, dark brown needle-shaped crystallites were obtained. Compound **3** afforded a molecular formula of C_15_H_15_NO_6_, as evidenced by negative HRESIMS (*m/z* 306.0981 [M − H]^−^, calculated 306.0972), with an index of hydrogen deficiency (IHDs) of nine. The UV spectrum exhibited an absorption maximum of 229.8 nm (Figure S1). The ^1^H NMR spectrum (Table 1) displayed five aromatic protons at *δ*_H_ 8.03 (1H, *d*, *J* = 7.5), *δ*_H_ 7.68 (1H, *dd*, *J* = 9.0, 1.5), *δ*_H_ 7.28 (1H, *t*, *J* = 9.0), *δ*_H_ 7.24 (1H, *dd*, *J* = 9.0, 1.5), and *δ*_H_ 6.14 (1H, *d*, *J* = 7.5); one hydroxy group at *δ*_H_ 5.40 (1H, *s*); two oxygenated methines at *δ*_H_ 3.59 (1H, *m*) and *δ*_H_ 3.48 (1H, *m*); and one oxygenated methylene at *δ*_Ha_ 3.65 (1H, *d*, *J* = 11.5) and *δ*_Hb_ 3.42 (1H, overlap); and one methylene at *δ*_Ha_ 2.64 (1H, *dd*, *J* = 11.5, 5.0) and *δ*_Hb_ 1.85 (1H, *dd*, *J* = 13.0, 6.0). Using DEPT and HSQC spectra, fifteen carbon resonances displayed in the ^13^C-NMR (Table 1) were assigned to one ketone carbon (*δ*_C_ 177.4); eight olefinic carbons (*δ*_C_ 143.4, 143.3, 127.0, 126.5, 124.4, 118.8, 117.9, and 110.5); an oxygenated quaternary carbon (*δ*_C_ 93.1); three oxygenated tertiary carbons (*δ*_C_ 85.0, 82.8, and 63.2); and two methylenes (*δ*_C_ 60.9 and 43.7). The ^1^H-^1^H COSY correlation (Figure 2) of H-5 (*δ*_H_ 7.68)/H-6 (*δ*_H_ 7.30)/H-7 (*δ*_H_ 7.24) and H-2(*δ*_H_ 8.03)/H-3(*δ*_H_ 6.14), along with HMBC correlations (Figure 2) of H-5 to C-8*a* (*δ*_C_ 127.0) and C-4 (*δ*_C_ 177.4), H-6 to C-4*a* (*δ*_C_ 126.5) and C-8 (*δ*_C_ 143.4), H-7 to C-8*a*, H-3/C-4*a* and H-2 to C-4 and C-8*a* indicated the existence of the characteristic 8-substituted-4-quinolone. Considering the remaining two unassigned IHDs, the rest of the group contained two rings. A tetrahydropyran ring was furnished by the HMBC correlations from H-11 (*δ*_H_ 2.46, 1.85) to C-9 (*δ*_C_ 85.0) and C-10 (*δ*_C_ 93.1) and from H-9 (*δ*_H_ 5.40) to C-13 (*δ*_C_ 82.8), in combination with the ^1^H-^1^H COSY correlations of H-11/H-12 (*δ*_H_ 3.85) and H-12/H-13 (*δ*_H_ 3.85). The key HMBC interactions of H-2/C-9 and H-9/C-8*a* and the downfield shifts of C-8, C-9, and C10 indicated that N-1, C-8*a*, C-8, C-9, and C-10 generated a morpholine ring. Furthermore, the ^1^H-^1^H COSY correlation H-13/H-14 (*δ*_H_ 3.67, 3.42) confirmed the connection between C-13 and C-14. Thus, the 2D structure of compound **3** was established. The relative configuration of compound **3** was confirmed by the ROESY spectrum (Figure 2), key correlations between H-9 and H-13, and between H-11 and H-13; and no correlation between H-12 and H-13 suggested that H-9 and H-13 were on the same side, but H-12 was on the opposite side. Therefore, the relative configuration of compound **3** could be 9*R*′,10*S*′,12*S*′,13*R*′. The absolute configuration of compound **3** was confirmed by ECD measurement and comparison with the calculated TD-DFT ECD data through the structure optimization by functional analysis of ωB97XD with the TZVP basis set and MeOH as SMD. The ECD calculation was performed after the optimization of conformers 5-a and 5-b. The comparison of the experimental and theoretically calculated ECD curves (Figure 3) confirmed the assignment of the absolute configuration of compound **3** as 9*R*,10*S*,12*S*,13*R*. The absolute configuration of compound **3** was also established by single-crystal X-ray diffraction with a suitable Flack parameter of −0.04 (Figure 4). The absolute configuration of **3** was determined to be 9*R*,10*S*,12*S*,13*R*, and it was named Indusiataine C.

Compound **4** was purified as a yellowish oil, and its molecular formula, C_13_H_20_O_3_, was determined by positive HRESIMS (*m/z* 225.1482 [M + H]^+^, calculated 225.1485) with an index of hydrogen deficiency (IHDs) of four. The UV spectrum of compound **4** displayed an absorption maximum of 277.1 nm (Figure S1). The ^1^H NMR spectrum (Table 1) indicated the presence of two *trans*-conjugated double bonds, as supported by hydrogen signals at *δ*_H_ 7.23 (1H, *d*, *J* = 11.5), *δ*_H_ 6.58 (1H, *dd*, *J* = 15.0, 11.5), and *δ*_H_ 6.37 (1H, *d*, *J* = 15.0). Additionally, two methylenes at *δ*_H_ 2.92 (2H, *t*, *J* = 6.0) and *δ*_H_ 2.66 (2H, *t*, *J* = 6.0); four methyls at *δ*_H_ 2.12 (3H, *s*), *δ*_H_ 1.79 (3H, *s*), and *δ*_H_ 1.24 (6H, *s*) were observed in the ^1^H NMR spectrum as well as active hydrogen signals at *δ*_H_ 4.86 (1H, *s*). The ^13^C-NMR spectrum revealed the resonances of 13 carbons (Table 1). Based on the DEPT spectrum, these resonances included two ketone carbons (*δ*_C_ 207.9 and *δ*_C_ 200.1), four olefinic carbons (*δ*_C_ 152.4, *δ*_C_ 139.1, *δ*_C_ 134.3, and *δ*_C_ 122.0), one quaternary carbon (*δ*_C_ 70.0), two methylenes (*δ*_C_ 37.5 and *δ*_C_ 31.4), and four methyl carbons (*δ*_C_ 30.3, *δ*_C_ 30.2, *δ*_C_ 30.2, and *δ*_C_ 12.0). The degree of unsaturation of compound **4** was calculated as 4, indicating that it had a linear structure. In the HMBC spectrum (Figure 2), correlations of H-1 (*δ*_H_ 2.12), H-3 (*δ*_H_ 2.66), H-4 (*δ*_H_ 2.92) to C-2 (*δ*_C_ 207.9) and correlations of H-3, H-4, and H-7 (*δ*_H_ 2.92) to C-5 (*δ*_C_ 200.1) were observed; the conjugated diene at H-7, H-8, and H-9 along with correlations of H-8 (*δ*_H_ 6.58)/C-6 (*δ*_C_ 134.3), H-9 (*δ*_H_ 6.37)/C-7 (*δ*_C_ 139.1), and H-8/C-10 (*δ*_C_ 70.0) and correlations of two methyls from H-11,12 (*δ*_H_ 1.24) to C-9 (*δ*_C_ 152.4) confirmed the two-dimensional (2D) structure of compound **4**. Thus, the structure of compound **4** was determined to be an acyclic norsesquiterpenoid and named Indusiataine D.

### 2.2. Biological Activity

Numerous fungal metabolites have been reported to have anti-inflammatory activities [34,35]. According to the structural information of these reported compounds and based on our research interests, compounds **1**, **3**, **4**, **11**, **15**, **16**, and **17** were selected for further biological evaluations.

#### 2.2.1. Effects of the Compounds on Cell Viability

**Figure 5:**
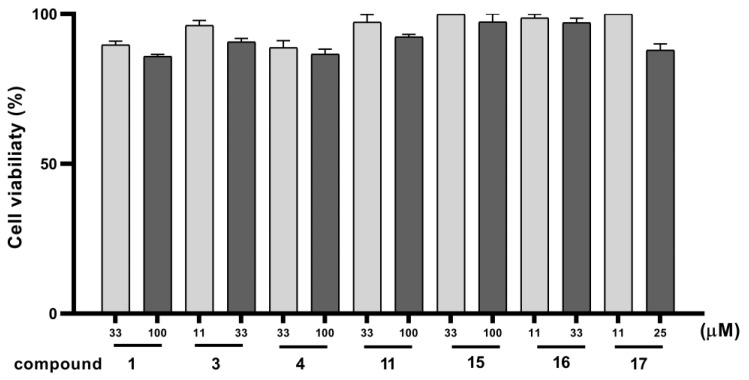
Effects of compounds **1**, **3**, **4**, **11**, **15**, **16**, and **17** at different concentrations on BV-2 cell viability. Cell viability was evaluated by thiazolyl blue tetrazolium bromide (MTT) assay. The results are represented by a percentage of examined viability of the normal cell group (where the cells were cultured under the normal growth condition), was set as 100%, and are expressed as the mean of triplicates; error bars indicate standard deviations (SD).

The cytotoxicity of compounds **1**, **3**, **4**, **11**, **15**, **16**, and **17** on BV-2 cells was evaluated using a thiazolyl blue tetrazolium bromide (MTT) assay [36]. As shown in Figure 5, after 24 h of exposure, the tested compounds at the examined concentrations did not induce significant cytotoxicity in the BV-2 cells (*p* > 0.05); therefore, subsequent in vitro anti-inflammatory experiments were performed with these concentrations.

#### 2.2.2. Effects of the Compounds on NO Production in LPS-Stimulated BV-2 Cells

During inflammation, the induced nitric oxide synthase (iNOS) would be activated and produce large amounts of nitric oxide (NO) [37]. The inhibitory effects of the selected compounds on NO production in LPS-induced microglia BV-2 cells were evaluated by measuring the nitrite content accumulated in the culture medium based on the Griess reaction [38]. Among the tested compounds (Table 2), compound **17** exhibited the most potent inhibitory effect against LPS-induced NO production in the BV-2 cells, with an IC_50_ of 10.86 μM, followed by another sterol compound, compound **15** (IC_50_: 42.41 μM). Compound **16** was the epimer of compound **15**; however, its IC_50_ value was 66.12 μM, indicating a comparatively weak inhibitory capacity. The IC_50_ values of three tested previously undescribed compounds, compounds **1**, **3**, and **4**, were 46.30 μM, 89.12 μM, and 62.15 μM, respectively. Moreover, compound **11** possessed no NO production inhibitory activity at the tested concentrations.

#### 2.2.3. Effects of the Compounds on Levels of TNF-α, IL-1β, and IL-6 in LPS-Stimulated BV-2 Cells

To examine the inhibitory effects of the selected compounds on the LPS-induced production of pro-inflammatory cytokines, we investigated the levels of tumor necrosis factor-α (TNF-α), interleukin-1β (IL-1β), and interleukin-6 (IL-6) in LPS-treated BV-2 cells using ELISA. As shown in Table 2, the treatments with compounds **1**, **3**, **11**, and **15** exhibited inhibitions of TNF-α production. Among them, compound **3** had the strongest inhibitory activity (IC_50_: 11.9 μM). The expression of LPS-induced IL-1β could be only inhibited by compound **17**, with an IC_50_ value of 23.9 μM. Regarding the LPS-induced generation of IL-6, the inhibitory effects of compounds **1**, **3**, **11**, **15**, and **16** were observed, and compound **16** displayed the lowest IC_50_ value of 13.53 μM.

#### 2.2.4. Effects of the Compounds on Expressions of iNOS and p-IκB-α in LPS-Stimulated BV-2 Cells

**Figure 6:**
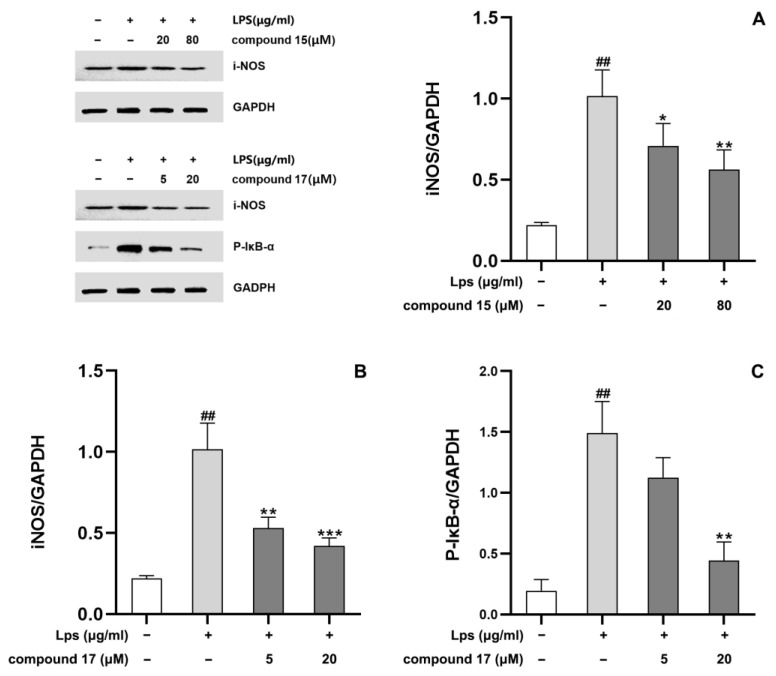
Significant alleviation of increased expression of inflammation-related proteins in lipopolysaccharide (LPS)-simulated BV-2 cells, following the treatments of compounds **15** and **17** with indicated concentrations. (**A**): compound **15** against iNOS expression. (**B**): compound **17** against iNOS expression. (**C**): compound **17** against *p*-IκB-α expression. The results are presented as the mean of triplicates; error bars indicate standard deviations. ## *p* < 0.01 for comparisons with the normal cell group, where the cells were cultured under the normal growth condition. * *p* < 0.05, ** *p* < 0.01, and *** *p* < 0.001 for comparisons with the LPS-treated group, where the cells were treated with LPS but without compounds.

A Western blot analysis was employed to further determine the inhibitory effects of the selected compounds on two key enzymes involved in the inflammatory response: iNOS and phosphorylated nuclear factor-kappa B inhibitor-α (*p*-IκB-α) [39]. Figure 6A,B show that compounds **15** and **17** significantly reduced iNOS expression at concentrations of 20 μM (*p* < 0.05) and 5 μM (*p* < 0.01), respectively. This finding is consistent with the results of the previous assay, where compounds **15** and **17** possessed the highest NO production inhibition activity. As shown in Figure 6C, compound **17** significantly inhibited *p*-IκB-α expression (*p* < 0.01). The treatments with the other tested compounds exhibited no or weak effects on the levels of iNOS and *p*-IκB-α, with no statistical differences (Figure S2).

### 2.3. Molecular Docking Simulation

A molecular docking study was conducted based on the results obtained from the previous in vitro experiments to further explore the anti-inflammatory mechanisms of the selected compounds.

**Figure 7:**
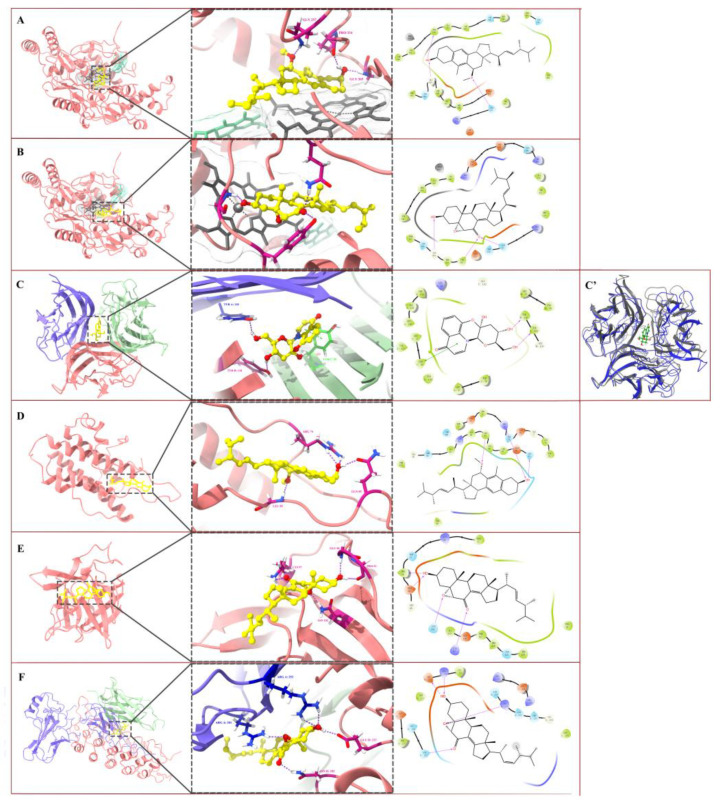
Molecular docking results of compounds **3**, **15**, **16**, and **17** with target inflammation-related proteins. (**A**): compound **15** with iNOS. (**B**): compound **17** with iNOS. (**C**) and (**C′**): compound **3** with TNF-α. (**D**): compound **16** with IL-6. (**E**): compound **17** with IL-1β. (**F**): compound **17** with IκB-α/NF-κB complex. For clarity, only interacting residues are labeled. In the three-dimensional representations, compounds are shown in the ball and stick model: yellow, carbon; red, oxygen; blue, nitrogen; white, hydrogen. Hydrogen bonding interactions are shown by purple dashes, and pi-interactions are shown by green dashes. In (**A**) and (**B**): black, haem molecule. In (**C**): purple, chain A; pink, chain B; cyan, chain C. In (**C′**): black, the original conformation of TNF-α; blue, the conformation of TNF-α formed after docking with compound **3**. In (**F**): purple, chain A; cyan, chain C; pink, chain D. In the two-dimensional representations, hydrogen bonding interactions are shown by purple arrows, arrows denote hydrogen bonds; pi-interactions are shown by green lines. Residues shown in green discs indicate hydrophobic interactions.

As compounds **15** and **17** exhibited good inhibitory activity on the LPS-induced generation of NO and the expression of iNOS in the BV-2 cells, a molecular docking study was performed to clarify their modes of interactions with iNOS. As shown in Figure 7A,B, the binding of compounds **15** and **17** with iNOS was achieved by stacking the compounds over the active region of haem and forming hydrogen bonds with the iNOS protein backbone [40]; both compounds fitted well within this pocket, with compound **15** forming three hydrogen bonds with Gln257, Pro334, and Gly365 (binding energy: −8.21 kcal/mol) and compound **17** establishing three hydrogen bonds with Glu257, Gly365, and Tyr367 (binding energy: −7.29 kcal/mol).

The compounds shown to have strong bio-activities in the ELISA tests were subjected to molecular docking studies with the corresponding pro-inflammatory cytokines. TNF-α has three identical receptor binding sites, as the protein possesses a unique threefold symmetry structure [41]. Compound **3** docked flexibly into the hydrophobic pocket located in the trimer core with a binding energy of −8.53 kcal/mol (Figure 7C). The three main binding interactions were the hydrogen bond established between the hydroxyl groups at C-14 with Tyr118 residues on chain A, the hydrogen bond established between the hydroxyl groups at C-12 with Tyr118 residues on chain B, and Pi-Pi stacking from the 4-pyridone ring with Tyr59 residues on chain C. Visible conformational changes in TNF-α occurred after the docking of compound **3**, the helices on chains A and B disappeared, and a large number of loop structures newly formed (Figure 7C′). Therefore, it was speculated that compound **3** inhibited TNF-α function by changing the protein structure and disrupting the receptor binding sites. Compound **16** bounded to two loop structures of IL-6 (Figure 7D) by forming three hydrogen bonds with Arg74, Leu89, and Gln85 (binding energy: −8.39 kcal/mol). Compound **17** matched well in the protein-binding pocket with IL-1β (Figure 7E); it formed four hydrogen bonds with Glu50, Pro51, Lys97, and Asn102 of IL-1β with a binding energy of −4.40 kcal/mol.

The IκB-α/NF-κB complex used for this molecular docking study is composed of three subunits [42]: NF-κB-P65 (chain A), NF-κB-P50 (chain C), and IκB-α (chain D) (Figure 7F). Compound **17** could be bound to the lower part of the central cavity of the complex and interacted with NF-κB-P65 (chain A) and IκB-α (chain D) (binding energy: −4.49 kcal/mol). The primary binding interactions were the hydrogen bonds formed between the hydroxyl group with Arg253 residues on chain A and Glu213 on chain D, the hydrogen bond established between the epoxy group and Arg201 on chain A, and the hydrogen bond formed between the carbonyl oxygen and Asn182 on chain D.

## 3. Discussion

### 3.1. Diversity of Metabolites with Small Molecular Weights in D. indusiata

There are four main stages in the morphological development of the fruit body of *D. indusiata*: the primordia stage, the ball-shaped stage, the peach-shaped stage, and the mature stage. An integrated quantitative proteomic and metabolomic analysis has revealed that [43], compared to the three early stages, more metabolites with <15 carbon atoms in the mature fruiting body of *D. indusiata* are upregulated, and for metabolites with ≥15 carbon atoms, more metabolites are downregulated. This phenomenon suggests that, in the final growth stage, large amounts of natural products with comparatively small molecular weights are preferentially biosynthesized and accumulated and that these chemical constituents may be crucial contributors to the taste, fragrance, and nutritional and medicinal properties of the mature fruiting body of *D. indusiata*. Our result is consistent with that reported above. In this research, eighteen small-molecule compounds were isolated from the ethanolic extract of *D. indusiata*, of which the number of core skeleton carbon atoms in **11** isolated compounds were less than **15**.

Among the four compounds newly described in this manuscript, three were terpenoids. Terpenoids are one of the largest groups of secondary metabolites found in nature. Edible fungi are considered prolific producers of structurally diverse terpenoid compounds, as these fungi possess an extensive repertoire of natural product biosynthesis pathways [44,45]. Compound **1** was a tricyclic sesquiterpenoid with a skeleton of antsorane [46,47], compound **2** was a rearranged cuparane-type sesquiterpenoid [48], and compound **4** was an acyclic norsesquiterpenoid [49,50]. The antsorane skeleton contained in compound **1** was quite unusual. As a natural product, antsorenone (synonym antsorane) was first discovered by Chazan in 1969 from the plant *Elionurus tristis* [46]. Several analogs were successively identified from *Eremophila fraseri* [51], *Vetiveria nigritana* [52], and *Chrysopogon zizanioides* [53]. The structure of antsorane was fully characterized by Gabriel P. Garcia et al. in 2019 [47]. To the best of our knowledge, compound **1** is the second natural compound ever reported with this specific sesquiterpenic ketone skeleton and the first one isolated from fungi. In Garcia et al.’s article, the high stability of the antsorane molecule was found, as it was subjected to several Brönsted and Lewis acid treatments and all the attempts to transform it into zizaen-3-one following a reverse Wagner–Meerwein rearrangement under acidic conditions failed. Accordingly, Garcia et al. hypothesized that the antsorane skeleton was the final step of the biosynthesis pathway that started with acorane. In terms of the molecular structure of our compound **1**, a hydroxyl group was present on the bridged ring of the antsorane skeleton; its formation pathway and its biological role in the ecology and physiology of *D. indusiata* deserve further attention and investigation.

The structural diversity of fungal terpenoids contributes to functional diversity. Terpenoids are widely involved in every aspect of fungi growth, development, and stress responses [45]. Fungal terpenoids play biological signaling and defensive roles, as they often possess a volatile profile and a unique aromatic property [54]; they can mediate many ecological interactions between fungi and other organisms. In this research, we found that compound **1** had a considerable sour and smelly odor, and the newly described compound **3** had an aroma of wood mixed with caramel. Together with the known odorous compounds, such as nicotinic acid (**6**) and oxindole (**10**), these small-molecular-weight compounds are vital contributors to the distinctive fragrance of *D. indusiata*.

According to the classes of the enzyme involved in their biosynthesis, the major groups of fungal secondary metabolites can be classified as polyketides, non-ribosomal peptides, terpenes, and alkaloids [55]; they can serve as metabolic precursors for a wide range of other metabolites. Only a relatively small number of higher fungal species have been chemically investigated; fungal natural products represent a vast and largely untapped source of potentially potent new pharmaceuticals [56,57]. Among fungal species, the basidiomycetes are one of the most important producers of diverse bioactive secondary metabolites [58,59]. *Ganoderma lucidum* and *Poria cocos* are two well-known medicinal basidiomycetes. Based on the phytochemical reports, more than 270 secondary metabolites have been reported to be isolated from *Ganoderma lucidum* [60,61,62]; more than 140 terpenoids and more than 20 steroids have been identified in *Poria cocos* [63,64]. As reviewed by Habtemariam [1] and described in the Section 1, the small-molecule compounds identified from *D. indusiata* mainly include a few terpenoids and alkaloids. The chemodiversity of this edible and medicinal mushroom species has not been adequately characterized. In this study, various terpenoids, steroids, lactones, fatty acids, and heterocyclic compounds were identified. The present findings reveal that abundant small-molecular-weight metabolites with highly complicated and diverse structures exist in the mushroom *D. indusiata*. These small molecules may form the basis for the further exploitation of *D. indusiata* as a source of functional ingredients for the food, cosmetic, and pharmaceutical industries.

### 3.2. Ergostane-Type Steroids Isolated from D. indusiata and Their Anti-Inflammatory Activities

The scientific literature on the biological activities of small-molecule metabolites in *D. indusiata* remains very scarce. Only two derivatives of albaflavenone isolated from *D. indusiata* were evaluated for their anti-inflammatory activities; these two compounds showed moderate inhibitory effects on LPS-induced NO production and TNF-*α* secretion in BV-2 cells [9]. In this research, three tested ergostane-type steroids (**15**, **16**, and **17**) isolated from *D. indusiata* exhibited good anti-inflammatory activities. Ergosterol is the primary sterol in fungal membranes and presumably contributes to membrane fluidity and function [65]. Ergosterol and its related derivatives are metabolites essential for fungal growth, development, and adaptation to various stresses [66]. Ergosterol biosynthesis can be divided into three modules: mevalonate, farnesyl pyrophosphate (farnesyl-PP), and ergosterol biosynthesis [67]. Ergosterol and some of its biosynthetic intermediates are critical natural products of great economic value. In the pharmaceutical industry, ergosterol is a direct precursor of vitamin D2 and steroid drugs [68].

Pharmaceutical steroids, non-steroidal anti-inflammatory drugs (NSAIDs), and anti-histamines have been widely applied in anti-inflammatory therapies [69]. In some cases, the marketed anti-inflammatory drugs may not deliver a radical cure and may cause side effects [70,71]. Therefore, the search for novel and safe anti-inflammatory agents or lead compounds in nature has become the focus of biologists and chemists. The extensive efforts devoted to searching for promising ergostane-type steroid compounds with high anti-inflammatory activities in fungi and to elucidating their mechanisms of action have recently been well-reviewed [34,35,66]. In the present study, the best anti-inflammatory activities (reflected by the smallest IC_50_ values) were exerted by compound **15** against TNF-α production (IC_50_ = 65.5 μM), compound **16** against IL-6 production (IC_50_ = 13.53 μM), and compound **17** against NO and IL-1β production (IC_50_ = 10.86 μM and 23.9 μM, respectively). Moreover, the evident suppression of iNOS and *p*-IκB-α expressions by compound **17** was observed. Since the experimental models vary among the related research, it is not easy to appropriately compare our results to the data reported in the literature.

Nevertheless, comparing the data obtained within our in vitro anti-inflammatory assays and in silico analysis may provide new insights into the structure–activity relationship of isolated sterols. Compounds **15** and **16** were two ergosterol derivatives with a rearranged tetracyclic skeleton [72]; they were epimers, and the stereo configuration of their methoxy at C-11 was on the same face and on the opposite face to the hydroxyl group on their molecules, respectively (Figure 1). Compound **15** showed higher inhibitory activity on LPS-induced NO production than compound **16**. From the visualized results of molecular docking, the hydroxyl group of compound **15** formed a hydrogen bond with Gly365 of iNOS. In addition, the methoxy group, which was on the same face as the hydroxyl group, formed another hydrogen bond with Gln257. Notably, compound **17**, the most potent NO production inhibitor found in this research, also established hydrogen bonds with the amino acid residues of Gly365 and Gln257 of iNOS. It can be deduced that the methoxy and the hydroxyl groups located on the same face of the molecule conferred compound **15** the capacity to interact with Gly365 and Gln257 simultaneously, which was crucial for its inhibitory effect against iNOS. In contrast, compound **16** showed the strongest inhibitory activity against IL-6 production among the tested compounds. The structure of IL-6 contained four relatively long α-helices (A–D) and two short helices (S1 and S2) linked via loop structures [73]. The docking result demonstrated that the binding site of compound **16** was situated in the loop region; the hydroxyl group of compound **16** interacted with Arg74 on the loop structure connected to the short helix S1; and the methoxy group located on the opposite face of the molecule could establish a hydrogen bond with Leu89 on the loop structure attached to the long helix B, with this fulfilling the firm binding and inhibition of IL-6.

Compound **17** is an epoxy-7-sitosterol, and the epoxy and carbonyl groups appeared to be the key functional elements. In the molecular docking analysis, hydrogen bonds formed by the epoxy group and carbonyl group of compound **17** with the amino acid residues of IL-1β were observed. At the same time, the ergosterol derivatives without these two substituents (compounds **15** and **16**) exhibited no activity against IL-1β; these two substituents of compound **17** also played essential roles in combination with the IκB-α/NF-κB complex, as they could establish hydrogen bonds with the NF-κB-P65 (chain A) and IκB-α (chain D), respectively, to form an IκB-α/NF-κB-P65/inhibitor (compound **17**) complex. This increased the binding affinity of IκB-α and NF-κB-P65, suggesting that the external factor-stimulated phosphorylation and degradation of IκB-α could subsequently be inhibited to certain degrees. The release of NF-κB-P65 from IκB-α; and the following relocation of NF-κB-P65 from the cytoplasm to the nucleus were suppressed, implying that the activation of NF-κB would be prevented [39]. This inference can be supported by the present data from Western blots. Thus, we can conclude that compound **17** exerts an anti-inflammatory effect by affecting the NF-κB signaling pathway. This research was mainly carried out by performing experiments on in vitro cell models and computer simulations. The results cannot fully elucidate the tested compounds’ actual effects and mechanisms of action. In vivo studies and clinical trials are needed to confirm these findings.

## 4. Materials and Methods

### 4.1. Fungi Material

The mature fruiting bodies of *D. indusiata* were collected from the Yibin edible fungi cultivation base (28°364″ N; 104°867″ E), Sichuan, China. The collection was performed in September 2021. The voucher specimen (No. D.I-B002) was deposited in the Key Laboratory of National Forestry and Grassland Administration/Beijing for Bamboo & Rattan Science and Technology, Beijing, China.

### 4.2. Chemicals and Reagents

The chromatographically pure reagents were purchased from Fisher-Scientific (Pittsburgh, PA, USA), ultrapure water was obtained using a PALL Lab Water Purification System (Port Washington, NY, USA), and other analytically pure reagents were supplied by Sinopharm (Beijing, China).

Dulbecco’s modified Eagle’s minimum essential medium (DMEM), fetal bovine serum (FBS), bovine serum albumin (BSA), and penicillin/streptomycin solution were obtained from Life Technologies, Inc. (Grand Island, NY, USA). Lipopolysaccharide (LPS) (*Escherichia coli*, serotype 055:B5), 3-(4,5-dimethylthiazol-2-yl)-2,5-diphenyl tetrazolium bromide (MTT), and dimethyl sulfoxide (DMSO) were obtained from Sigma–Aldrich (St. Louis, MO, USA). Glyceraldehyde-3-phosphate dehydrogenase (GAPDH), primary antibodies against iNOS and *p*-IκB-α were obtained from Cell Signaling Technology (Danvers, MA, USA). The nitric oxide (NO) assay kit (S0021) and the mouse TNF-α, IL-6, and IL-1β ELISA kits (PT512, PI326, and PI301, respectively) were purchased from Beyotime Biochemical (Haimen, China).

### 4.3. Cell Culture

The mouse microglial cell line BV-2 was obtained from the Institute of Basic Medical Sciences, Chinese Academy of Medical Sciences (Beijing, China). The cells were cultured in DMEM supplemented with 10% FBS and 1% penicillin/streptomycin at 37 °C in a humidified incubator with an atmosphere of 5% CO_2_.

### 4.4. General Experimental Procedures

The isolation of the compounds was performed by using repeated open-column chromatography (CC), preparative thin-layer chromatography (TLC), and preparative high-performance liquid chromatography (HPLC). The chemical structure of the isolated compounds was determined by the spectra of analytical HPLC, UV, ^1^H and ^13^C-NMR, HRESIMS, CD, and XRD. Instrumental details were described in Supplementary Materials (Table S1).

### 4.5. Extraction and Isolation

After air-dried, 50 kg *D. indusiata* fruiting bodies were repeatedly extracted four times with 85% EtOH under reflux. The resulting extracts were combined and filtered (No.1 filter paper Whatman, Maidstone, England), then evaporated under reduced pressure using a rotary evaporator (R-215, Buchi, Flawil, Switzerland) to give an organic solvent-free crude extract: 12.60 kg. The crude extract was resuspended in water and then partitioned with EtOAc. The EtOAc-partitioned extract (800 g) was chromatographed on a silica gel column with gradient elution of CH_2_Cl_2_/MeOH (200:1, 100:1, 30:1, 10:1, 5:1, 2:1, 0:1, *v*/*v*) to give 28 fractions (ZS1–ZS28). The fractions with comparatively high yields, including ZS7 (9.7 g), ZS12 (13.2 g), ZS13 (6.3 g), ZS14 (7.6 g), ZS24 (50.6 g), and ZS27 (4.7 g), were subjected to the further separations. Fraction ZS7 was separated into five subfractions ZS7a–ZS7e on a silica gel column eluted with CH_2_Cl_2_/MeOH (5:1, 2:1, *v*/*v*) and MeOH. ZS7c (4.4 g) was submitted to a Sephadex LH-20 column, eluted with petroleum ether/CH_2_Cl_2_/MeOH (5:5:1, *v*/*v*), followed by a separation on preparative HPLC using eluting solvents of MeCN/H_2_O (95:5, *v*/*v*), MeOH/H_2_O (55:45, *v*/*v*), and MeOH, to obtain compound **13** (23.0 mg), compound **11** (6.1 mg) and compound **14** (1.5 g). Fraction ZS12 was subjected to a Sephadex LH-20 column eluted with a gradient system of petroleum ether/CH_2_Cl_2_/MeOH (10:10:1 to 1:1:3, *v*/*v*) and MeOH to yield 11 subfractions (ZS12a–ZS12k). ZS12i was proven to be a single compound **10** (58.00 mg) by HPLC analysis. Fraction ZS13 was separated on a Sephadex LH-20 column eluted with petroleum ether/CH_2_Cl_2_ (5:1, *v*/*v*) to yield six subfractions (ZS13a–ZS13f). The separation of ZS13a was conducted on a preparative TLC using petroleum ether/CH_2_Cl_2_/MeOH (7:7:1, *v*/*v*) as the elution solvent, followed by a preparative HPLC (MeOH/H_2_O, 80:20, *v*/*v*) to obtain compound **17** (4.8 mg) and compound **18** (2.9 mg). Same preparative thin layer chromatography separations were carried out on ZS13c, ZS13d, ZS13e, and ZS13f, and these four fractions were further separated by preparative HPLC eluted with MeOH/H_2_O (70:30, *v*/*v*), MeOH/H_2_O (70:30, *v*/*v*), MeOH/H_2_O (60:40, *v*/*v*), and MeOH/H_2_O (87:13, *v*/*v*), respectively, to obtain compound **4** (7.9 mg), compound **2** (2.9 mg), compound **1** (1.6 mg), and compound **7** (5 mg). Fraction ZS27 was separated using a Sephadex LH-20 column eluted with petroleum ether/CH_2_Cl_2_/MeOH (2:2:1, *v*/*v*) to yield nine subfractions (ZS27a–ZS27i). ZS27f was submitted to preparative TLC eluted with petroleum ether/CH_2_Cl_2_/MeOH (3:3:1, *v*/*v*) and further purified on a preparative HPLC (MeOH/H_2_O, 20:80, *v*/*v*) to obtain compound **12** (1.2 mg). ZS27d, ZS27h, and ZS27i were isolated by preparative HPLC with different elution solvents of MeOH/H_2_O (55:45, *v*/*v*), MeOH/H_2_O (31:69, *v*/*v*), and MeOH/H_2_O (36:64, *v*/*v*), to afford compound **5** (1.5 mg), compound **3** (12 mg), and compound **9** (15 mg), respectively. Fraction ZS24 was purified on a silica gel column, eluted with a gradient of CH_2_Cl_2_/MeOH (6:1 to 0:1, *v*/*v*), and further isolated by preparative HPLC (MeOH/H_2_O, 10:90, *v*/*v*) to give compound **6** (50 mg). Fraction ZS14 was also chromatographed on a Sephadex LH-20 column eluted with petroleum ether/CH_2_Cl_2_/MeOH (5:5:1, *v*/*v*) to yield five subfractions (ZS14a to ZS14e). ZS14b and ZS14d were further separated using a Sephadex LH-20 column eluted with petroleum ether/CH_2_Cl_2_ (1:1, *v*/*v*), then subjected to a preparative HPLC eluted with MeOH to obtain compound **15** (3.5 mg), compound **16** (7.7 mg), and compound **8** (2.7 mg).

Characterization data of previously undescribed compounds:

Compound **1**: yellowish amorphous powder, UV(MeOH) λ_max_ 248.7 nm; HRESIMS *m/z*: 235.1690 [M + H]^+^ (calcd for C_15_H_22_O_2_, 235.1693). ^1^H NMR data (DMSO-*d*_6_, 500 MHz) and ^13^C-NMR data (DMSO-*d*_6_, 125 MHz) are shown in Table 1. CD spectrum (MeOH) is shown in Figure 3.

Crystal data: C_15_H_22_O_2_, *M* = 234.32, orthorhombic, *a* = 7.4315(7) Å, *b* = 13.1582(12) Å, *c* = 26.367(2) Å, *U* = 2578.3(4) Å^3^, *T* = 111.6(9), space group C222_1_ (no. 20), *Z* = 8, *μ*(Cu K*α*) = 0.612, 4187 reflections measured, 2412 unique (*R*_int_ = 0.0675) which were used in all calculations. The final *wR*(*F*_2_) was 0.1293.

Compound **2**: yellowish oil, UV(MeOH) λ_max_ 235.7 nm; HRESIMS *m/z*: 235.1694 [M + H]^+^ (calcd for C_15_H_22_O_2_, 235.1693). ^1^H NMR data (DMSO-*d*_6_, 500 MHz) and ^13^C-NMR data (DMSO-*d*_6_, 125 MHz) are shown in Table 1. CD spectrum (MeOH) is shown in Figure 3.

Compound **3**: brown crystal, UV(MeOH) λ_max_ 229.8 nm; HRESIMS *m/z*: 306.0981 [M + H]^−^ (calcd for C_15_H_15_NO_6_, 306.0972). ^1^H NMR data (DMSO-*d*_6_, 500 MHz) and ^13^C-NMR data (DMSO-*d*_6_, 125 MHz) are shown in Table 1. CD spectrum (MeOH) is shown in Figure 3.

Crystal data: C_15_H_15_NO_7_, *M* = 321.28, trigonal, *a* = 26.3585(15) Å, *c* = 5.4846(4) Å, *U* = 3300.1(4) Å^3^, *T* = 113(2), space group R3 (no. 146), *Z* = 9, *μ*(Cu K*α*) = 0.998, 5982 reflections measured, 2699 unique (*R*_int_ = 0.0302), which were used in all calculations. The final *wR*(*F*_2_) was 0.1274.

Compound **4**: yellowish oil, UV(MeOH) λ_max_ 277.1 nm; HRESIMS *m/z*: 225.1482 [M + H]^−^ (calcd for C_13_H_20_O_3_, 225.1485). ^1^H NMR data (DMSO-*d*_6_, 500 MHz) and ^13^C-NMR data (DMSO-*d*_6_, 125 MHz) are shown in Table 1. CD spectrum (MeOH) is shown in Figure 3.

### 4.6. In Vitro Anti-Inflammatory Assay

Compounds **1**, **3**, **4**, **11**, **15**, **16**, and **17** were selected for in vitro investigations of anti-inflammatory activities using an LPS-stimulated BV-2 microglial cell model. Three groups were designed for the experiments: the normal cell group, where the cells were cultured under normal growth conditions; the LPS group, where the cells were treated with LPS but without the tested compounds; and the sample group, where the cells were treated with LPS plus the tested compounds.

#### 4.6.1. MTT Assay for the Measurement of Cell Viability

The cytotoxic effects of the selected compounds on the BV-2 cells were evaluated using an MTT assay [38]. Briefly, BV-2 cells were seeded into 96-well plates at a density of 8 × 10^3^ cells/well and cultured overnight in an incubator at 37 °C with 5% CO_2_. Then, the cells were treated with different concentrations of the selected compounds diluted with DMEM. After 2 h, LPS (1 μg/mL) was added. After 24 h of incubation, 20 μM MTT was added to each well and the cells were incubated at 37 °C for 4 h. Afterward, the supernatant was discarded, and 150 μL of DMSO was added to each well to dissolve the formazan. The absorbance (optical density, OD) of each well was measured at 490 nm using a multilabel plate reader (Victor 2030, PerkinElmer, Waltham, MA, USA). The cell viability results were calculated as percentages.

#### 4.6.2. Measurement of Nitric Oxide and Cytokine Production

The tested compounds were diluted in a gradient with DMEM. The BV-2 cells were seeded into 96-well plates at a density of 8 × 10^3^ cells/well, cultured overnight in an incubator at 37 °C with 5% CO_2_, and then cultured with LPS (1 μg/mL) in the absence or presence of the tested compounds for 24 h. The culture supernatants were collected for the measurements. The total nitric oxide level was measured using the NO assay kit. The TNF-α, IL-1β, and IL-6 concentrations were quantified using mouse ELISA kits. All operations were conducted according to the manufacturer’s instructions.

#### 4.6.3. Western Blot Analysis

The protein expressions of iNOS and *p*-IκB-α were measured using a Western blot analysis [74]. The tested-compound-treated BV-2 cells in the logarithmic growth phase were digested in a single-cell suspension with trypsin and seeded into 6-well plates at a density of 3 × 10^5^ cells/well. Then, the cells were washed twice with 4 °C phosphate-buffered saline (PBS) and harvested. Next, 200 μL of radioimmunoprecipitation assay (RIPA) buffer was added to each well, and the lysate was centrifuged at 12,000× *g* for 20 min after incubation at 4 °C for 30 min. The protein concentration was determined using a bicinchoninic acid (BCA) assay. The protein was collected for Western blot analyses of iNOS and *p*-IκB-α. After being resolved using SDS/PAGE loading buffer, the protein was electrophoretically separated at 120 V for 90 min and transferred to polyvinylidence difluoride (PVD) membranes at 300 mA for 85 min. Then, the PVD membranes were blocked with 5% skim milk in phosphate-buffered saline containing 0.05% Tween-20 (PBST) for 120 min at room temperature. The cells were incubated overnight at 4 °C in diluted primary antibodies (anti-iNOS and anti-*p*-IκB-α). The membrane was washed with PBST three times and incubated in a (horseradish peroxidase) HRP-conjugated secondary antibody solution for 120 min at room temperature. Finally, an appropriate amount of enhanced chemiluminescence reagent was added to detect the protein. The data for iNOS and *p*-IκB-α were normalized on the basis of GAPDH levels.

### 4.7. Molecular Docking

The protein structures of TNF-α (PDB ID: 7KP8), IL-1β (PDB ID: 8I1B), iNOS (PDB ID: 4UX6), and IL-6 (PDB ID: 2L3Y) were downloaded from the Protein Data Bank (http://www.rcsb.org/, accessed on 1 February 2023). The structure of the IκB-α/NF-κB complex (PDB ID: 1IKN) was modeled using SwissModel (http://swissmodel.expasy.org/, accessed on 1 February 2023) to remove the repeats of subunits from the heterodimer [42]. Each structure was prepared and refined using the Protein Preparation Wizard of Maestro 11.9 (Schrodinger LLC, New York, NY, USA). Hydrogens were added, water molecules were removed, energy was optimized, and the force-field parameters were adjusted to make them a low-energy conformation satisfying the ligand structure [75]. The amino acids were modified with a flexible setting, and the polar hydrogen atoms of the specific amino acids in the binding pocket were allowed to rotate during the optimization of the docking poses. The 3D structures of compounds **3**, **15**, **16**, and **17** were created and optimized to lower-energy conformers with the Maestro Build Panel, and they were used as the ligands for the molecular docking simulations. The glide ligand docking and induced-fit docking modules in Maestro 11.9 software were employed for the docking study and for the analysis of the results [76,77]. The docking results were visualized using USCF ChimeraX (https://www.rbvi.ucsf.edu/chimerax, accessed on 1 February 2023).

### 4.8. Statistical Analysis

Each experiment was repeated three times, with three replicates within each experiment. The data are expressed as mean ± standard deviations (SD). IBM SPSS software (version 23.0, SPSS Inc. Chicago, IL, USA) was used to generate graphs and perform statistical analyses. Data were analyzed using a Student’s *t*-test and one-way analysis of variance (ANOVA). A *p*-value < 0.05 was considered statistically significant.

## 5. Conclusions

A chemical investigation of the ethyl acetate fraction of *D. indusiata* ethanolic extract resulted in the isolation and identification of 18 compounds (**1**–**18**), including three previously undescribed sesquiterpenoids (**1**, **2**, and **4**) and one previously undescribed quinolone derivate (**3**). Their structures and absolute configurations were elucidated using spectroscopic methods and chemical analyses. Among them, compound **1** was the first natural product identified from fungi with an unusual molecular skeleton of antsorane. Seven isolated compounds were evaluated for anti-inflammatory activities in an LPS-stimulated BV-2 microglial cell model. Compound **3** had the best inhibitory effect on TNF-α secretion. Compound **16**, an ergosterol derivative with a rearranged tetracyclic skeleton, showed the most potent activity on the suppression of IL-6 generation. The 5α,6α-epoxy-7-sitosterol compound **17** exhibited an anti-inflammatory effect by inhibiting the production of NO and IL-1β and the expressions of iNOS and *p*-IκB-α. The molecular docking study conducted in this research indicated that the tested compounds could exert their anti-inflammatory activities by binding to the hydrophobic pocket of the corresponding protein. These findings suggest that, as *D. indusiata* is an edible and medicinal mushroom, its anti-inflammatory property would be at least partially contributed by the presence of a series of compounds with small molecular weights as active constituents. The results of this study demonstrate the chemodiversity and biological potential of the small-molecule metabolites in *D. indusiata*. They provide a foundation for future investigations into the molecular basis of the pharmacological effects of the mushroom *Dictyophora indusiata*.

## Figures and Tables

**Table 1:** ^1^H and ^13^C-NMR spectroscopic data (DMSO-*d*6, 500/125 MHz) for compounds **1**–**4**.

	Compound 1			Compound 2		Compound 3			Compound 4		
no	*δ*_C_	*δ*_H_, multi., *J*	no	*δ*_C_	*δ*_H_, multi., *J*	no	*δ*_C_	*δ*_H_, multi., *J*	no	*δ*_C_	*δ*_H_, multi., *J*
1	132.9		1	206.8		2	143.3	8.03, *d*, 7.5	1	30.3	2.12, *s*
2	208.4		2	129.3	5.88, *s*	3	110.5	6.14, *d*, 7.5	2	207.9	
3	44.7	2.08, 1.86, *d*, 18.0	3	193.5		4	177.4		3	37.5	2.66, *t*, 6.0
4	43.6	1.14, *m*	4	44.9		4a	126.5		4	31.4	2.92, *t*, 6.0
5	34.0		5	54.8	2.25, *d*, 11.0	5	117.9	7.68, *dd*, 9.0, 1.5	5	200.1	
6	46.8	1.36, *m*	6	28.5	1.34, *s*	6	124.4	7.28, *t*, 9.0	6	134.3	
7	65.1	4.82, *s*	7	29.4	1.26, *s*	7	118.8	7.24, *dd*, 9.0, 1.5	7	139.1	7.23, *d*, 11.5
8	181.5		1′	41.4		8	143.4		8	122.0	6.58, *d*, 15.0, 11.5
9	45.2		2′	132.9	5.72, *dd*, 10.0, 1.0	8a	127.0		9	152.4	6.37, *d*, 15.0
10	25.4	1.91, 0.98, *m*	3′	135.1	5.56, *dd*, 10.0, 1.0	9	85.0	5.40, *s*	10	70.0	
11	16.6	1.78, 1.58, *m*	4′	66.2		10	93.1		11	30.2	1.24, *s*
12	8.5	1.69, *s*	5′	35.3	1.61, 1.40, *m*	11	43.7	2.64, *dd*, 11.5, 5.0; 1.85, *dd*, 13.0, 6.0	12	12.0	1.79, *s*
13	11.6	0.86, *d*, 7.0	6′	34.3	1.92, 1.74, *m*	12	63.2	3.59, *m*	13	30.2	1.24, *s*
14	30.3	0.94, *s*, 1.5	7′	29.9	1.28, *s*	13	82.8	3.48, *m*			
15	25.2	0.94, s, 1.5	8′	29.5	1.10, *s*	14	60.9	3.65, *d*, 11.5; 3.42, overlap			
7-OH		5.19, *d*, 6.0	4′-OH		4.55, *s*						

DMSO-*d*6: deuterated dimethyl sulfoxide. Internal reference standard: tetramethylsilane (TMS). Chemical shifts (*δ*) are recorded in parts per million (ppm), and coupling constants (*J*) are given in Hz.

**Table 2:** In vitro inhibitory activities of compounds **1**, **3**, **4**, **11**, **15**, **16**, and **17** against LPS-stimulated production of pro-inflammatory mediators in BV-2 cells.

Compounds	Pro-Inflammatory Mediators (IC_50_ in µM)			
	NO	TNF-α	IL-1β	IL-6
**1**	46.3 ± 1.35	34.5 ± 2.1	>100	77.57 ± 4.43
**3**	89.12 ± 8.54	11.9 ± 1.25	>100	46.34 ± 2.34
**4**	62.15 ± 4.64	>100	>100	>100
**11**	>100	86.2 ± 6.33	>100	58.43 ± 2.23
**15**	42.41 ± 3.44	65.5 ± 1.45	>100	49.43 ± 2.15
**16**	66.12 ± 4.38	>100	>100	13.53 ± 1.46
**17**	10.86 ± 0.73	>100	23.9 ± 1.48	>100

Results are presented as 50% inhibitory concentration (IC_50_) values in μM. The LPS-stimulated production of pro-inflammatory mediators in the LPS-treated group (where the cells were treated with LPS but without compounds) was set as 100%. All measurements were carried out in triplicates. Data are mean value ± standard deviation. LPS: lipopolysaccharide. NO: nitric oxide. TNF-α: tumor necrosis factor-α. IL: interleukin.

## Data Availability

Crystallographic data for compounds **1** and **3** have been deposited at the Cambridge Crystallographic Data Centre (www.ccdc.cam.ac.uk/conts/retrieving.html, accessed on 1 February 2023) with codes 2157828 (**1**) and 2167830 (**3**), respectively.

## Supplementary Materials

The following supporting information can be downloaded at: https://www.mdpi.com/article/10.3390/molecules28062760/s1, Figure S1: Spectra of compounds **1**–**4**; Figure S2: Effects of tested compounds on expression of inflammation-related proteins; Table S1: Main experimental instruments.

## Sample Availability

Samples of the compounds are not available from the authors.
