# Supplementary material for: Chemical Constituents of the Mushroom Dictyophora indusiata and Their Anti-Inflammatory Activities
Source: Molecules. 2023 Mar 18;28(6):2760. doi: 10.3390/molecules28062760 (PMC10052543; doi:10.3390/molecules28062760)
Supplement: Supplementary file 1 [file molecules-28-02760-s001.zip › molecules-2270402-supplementary.pdf]

Supporting Information

Contents

- **Figure S1.** Spectra of compounds **1-4**.

Figure S1 A-1. UV spectrum of compound **1**

Figure S1 A-2. HRESIMS spectrum of compound **1**

Figure S1 A-3. ^{1}H NMR spectrum of compound **1**

Figure S1 A-4. ^{13}C NMR spectrum of compound **1**

Figure S1 A-5. DEPT-90 spectrum of compound **1**

Figure S1 A-6. DEPT-135 spectrum of compound **1**

Figure S1 A-7. ^{1}H-^{1}H COSY spectrum of compound **1**

Figure S1 A-8. HMBC spectrum of compound **1**

Figure S1 A-9. HSQC spectrum of compound **1**

Figure S1 A-10. ROESY spectrum of compound **1**

Figure S1 B-1. UV spectrum of compound **2**

Figure S1 B-2. HRESIMS spectrum of compound **2**

Figure S1 B-3. ^{1}H NMR spectrum of compound **2**

Figure S1 B-4. ^{13}C NMR spectrum of compound **2**

Figure S1 B-5. DEPT-90 spectrum of compound **2**

Figure S1 B-6. DEPT-135 spectrum of compound **2**

Figure S1 B-7. ^{1}H-^{1}H COSY spectrum of compound **2**

Figure S1 B-8. HMBC spectrum of compound **2**

Figure S1 B-9. HSQC spectrum of compound **2**

Figure S1 B-10. ROESY spectrum of compound **2**

Figure S1 C-1. UV spectrum of compound **3**

Figure S1 C-2. HRESIMS spectrum of compound **3**

Figure S1 C-3. ^{1}H NMR spectrum of compound **3**

Figure S1 C-4. ^{13}C NMR spectrum of compound **3**

Figure S1 C-5. DEPT-90 spectrum of compound **3**

Figure S1 C-6. DEPT-135 spectrum of compound **3**

Figure S1 C-7. ^1H-^1H COSY spectrum of compound **3**

Figure S1 C-8. HMBC spectrum of compound **3**

Figure S1 C-9. HSQC spectrum of compound **3**

Figure S1 C-10. ROESY spectrum of compound **3**

Figure S1 D-1. UV spectrum of compound **4**

Figure S1 D-2. HRESIMS spectrum of compound **4**

Figure S1 D-3. ^1H NMR spectrum of compound **4**

Figure S1 D-4. ^{13}C NMR spectrum of compound **4**

Figure S1 D-5. DEPT-90 spectrum of compound **4**

Figure S1 D-6. DEPT-135 spectrum of compound **4**

Figure S1 D-7. HMBC spectrum of compound **4**

Figure S1 D-8. HSQC spectrum of compound **4**

- **Figure S2.** Effects of tested compounds on expression of inflammation-related proteins.

Figure S2 A. Effects of compound **1** on expression of iNOS and *p*-I*κ*B-*α* in lipopolysaccharide (LPS)-simulated BV-2 cells.

Figure S2 B. Effects of compounds **3** and **4** on expression of iNOS and *p*-I*κ*B-*α* in lipopolysaccharide (LPS)-simulated BV-2 cells.

Figure S2 C. Effects of compound **11** on expression of iNOS and *p*-I*κ*B-*α* in lipopolysaccharide (LPS)-simulated BV-2 cells.

Figure S2 D. Effects of compound **15** on expression of *p*-I*κ*B-*α* in lipopolysaccharide (LPS)-simulated BV-2 cells.

Figure S2 E. Effects of compound **16** on expression of iNOS and *p*-I*κ*B-*α* in lipopolysaccharide (LPS)-simulated BV-2 cells.

- **Table S1.** Main experimental instruments.

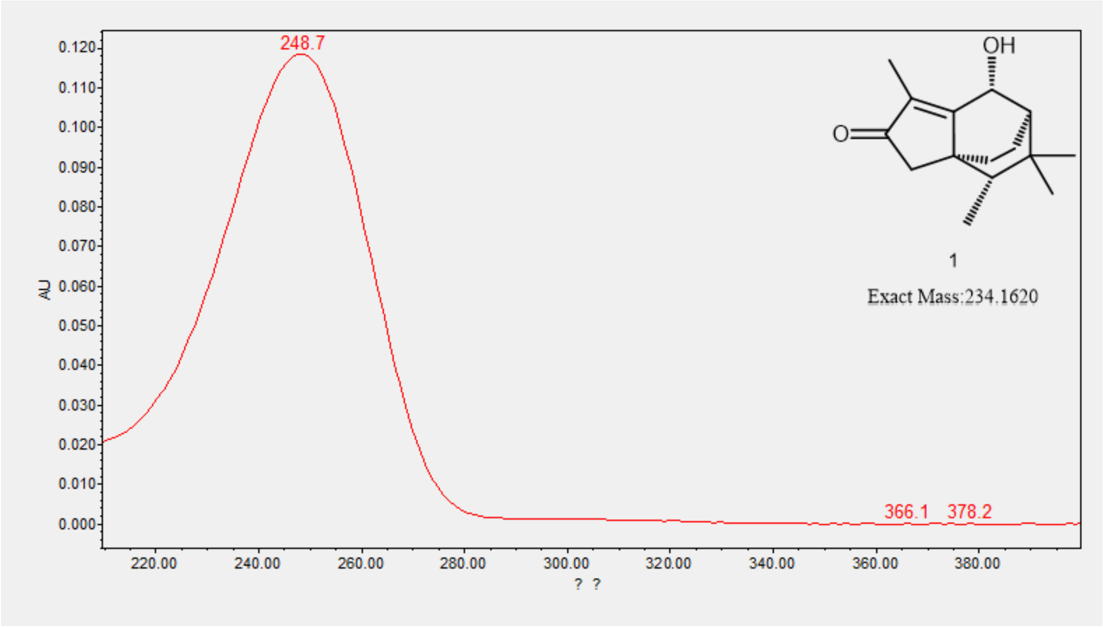

Figure S1 A-1. UV spectrum of compound **1**

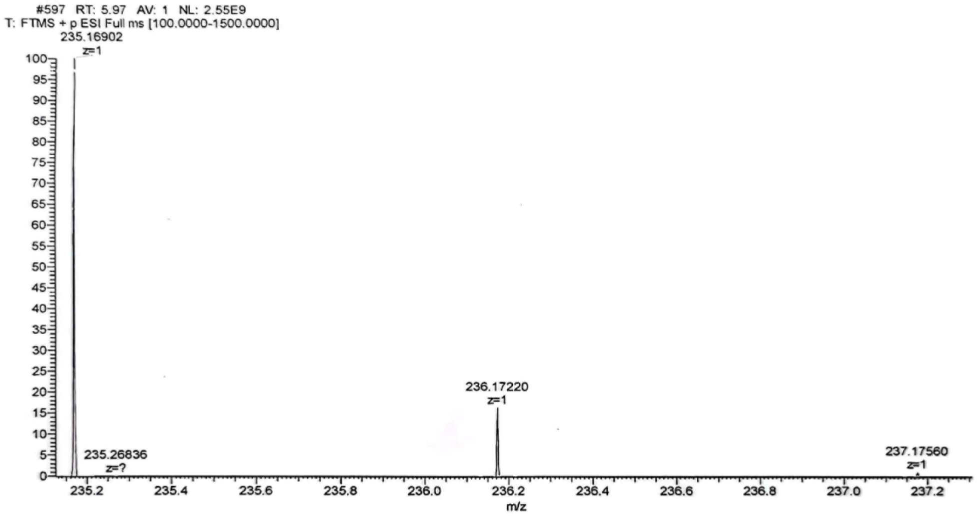

Figure S1 A-2. HRESIMS spectrum of compound **1**

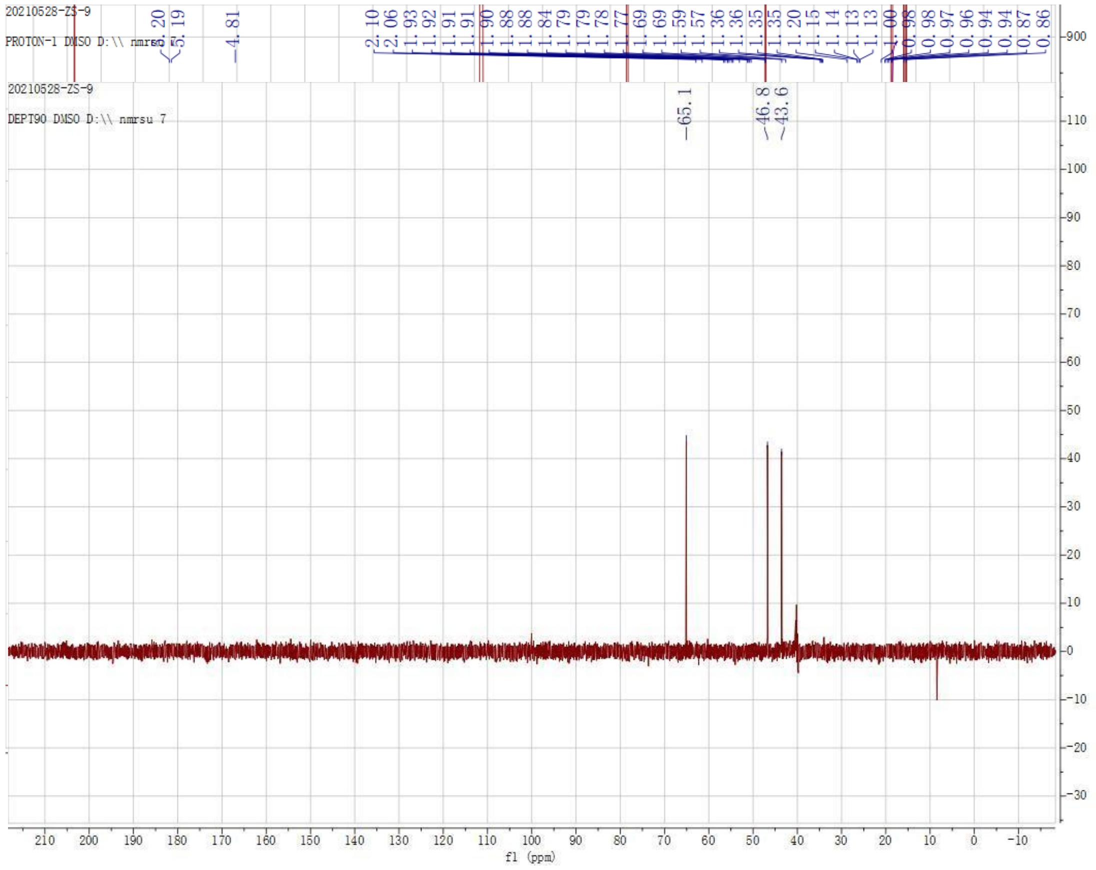

Figure S1 A-3. ^{1}H NMR spectrum of compound **1**

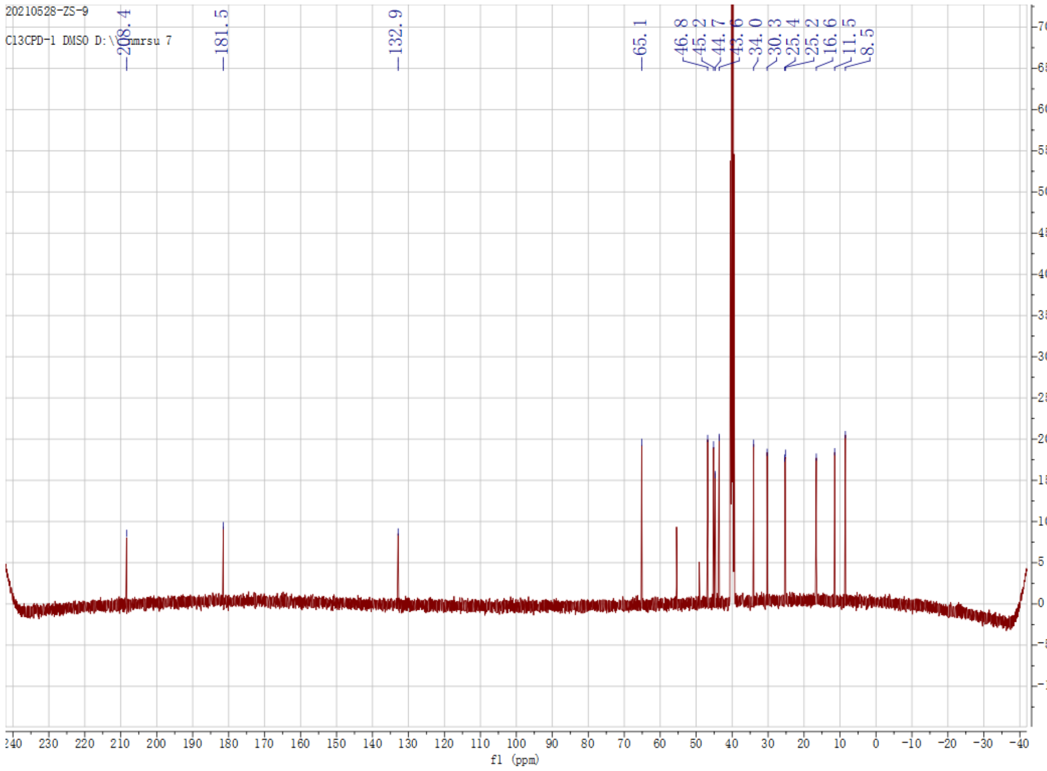

Figure S1 A-4. ^{13}C NMR spectrum of compound **1**

Figure S1 A-5. DEPT-90 spectrum of compound **1**

Figure S1 A-6. DEPT-135 spectrum of compound **1**

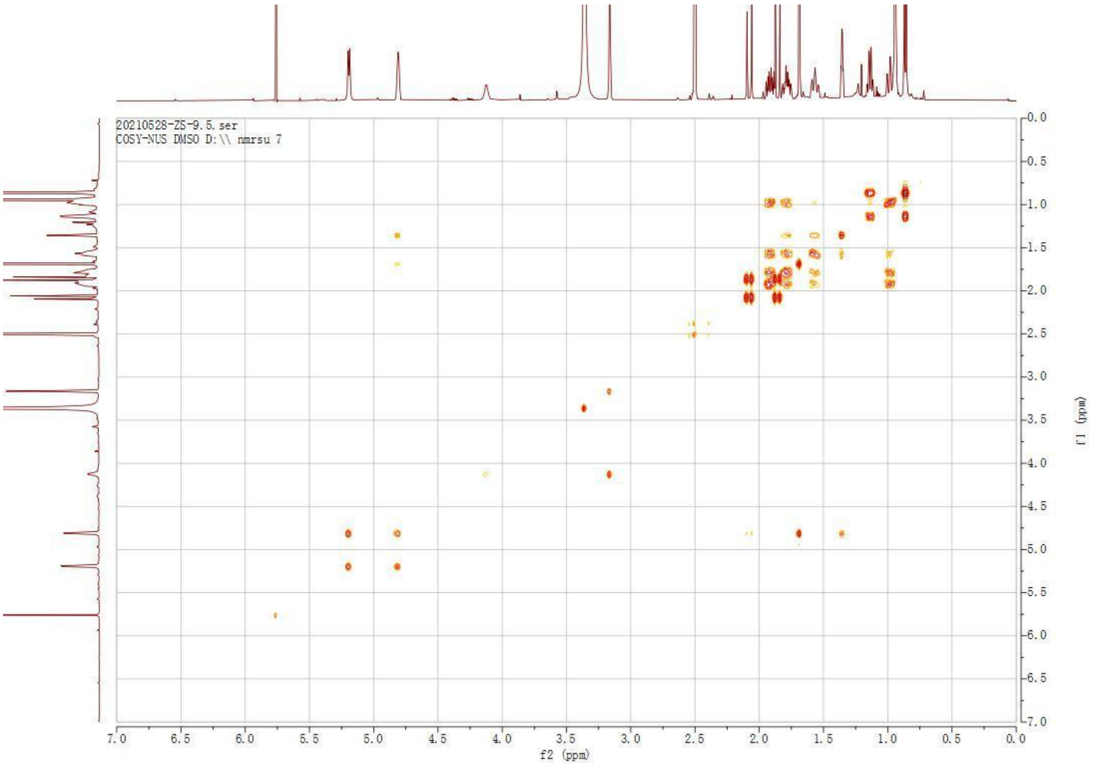

Figure S1 A-7. ^{1}H-^{1}H COSY spectrum of compound **1**

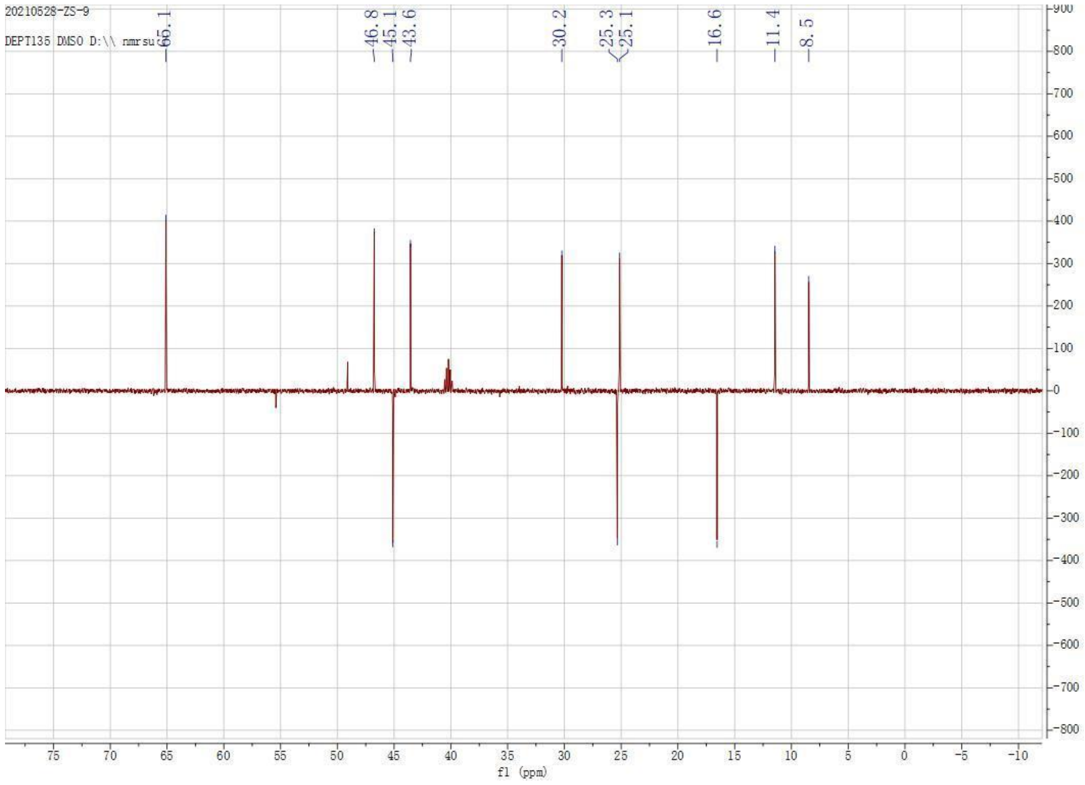

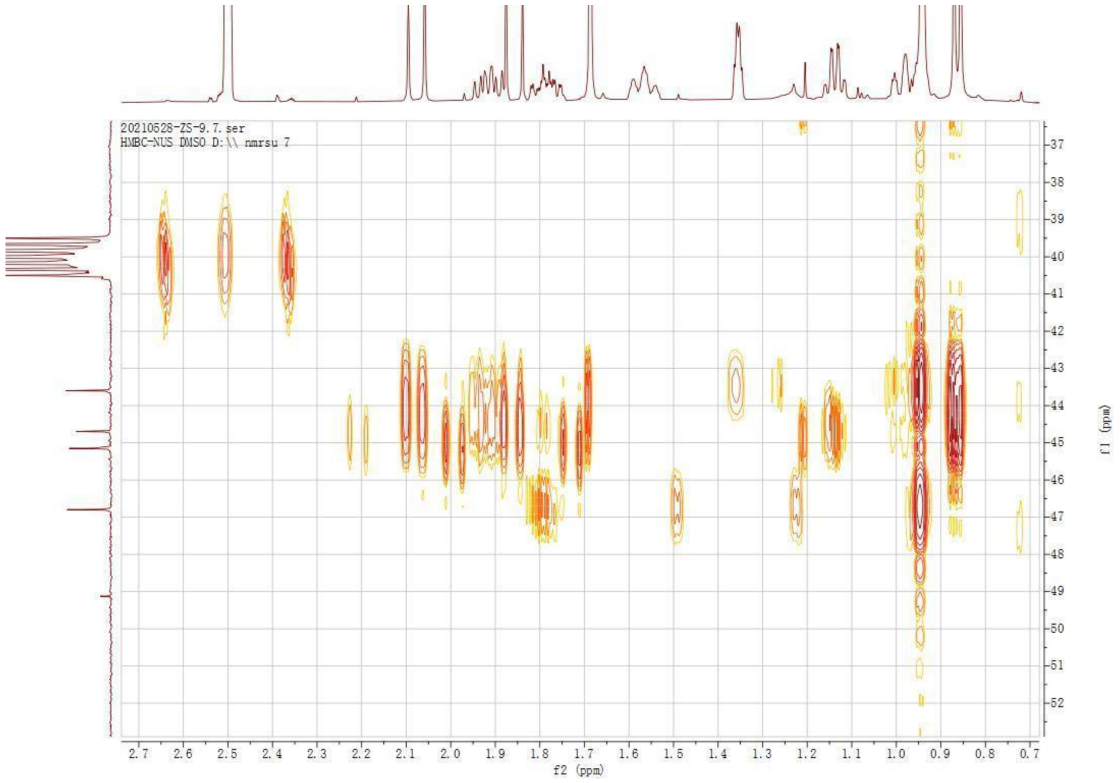

Figure S1 A-8. HMBC spectrum of compound **1**

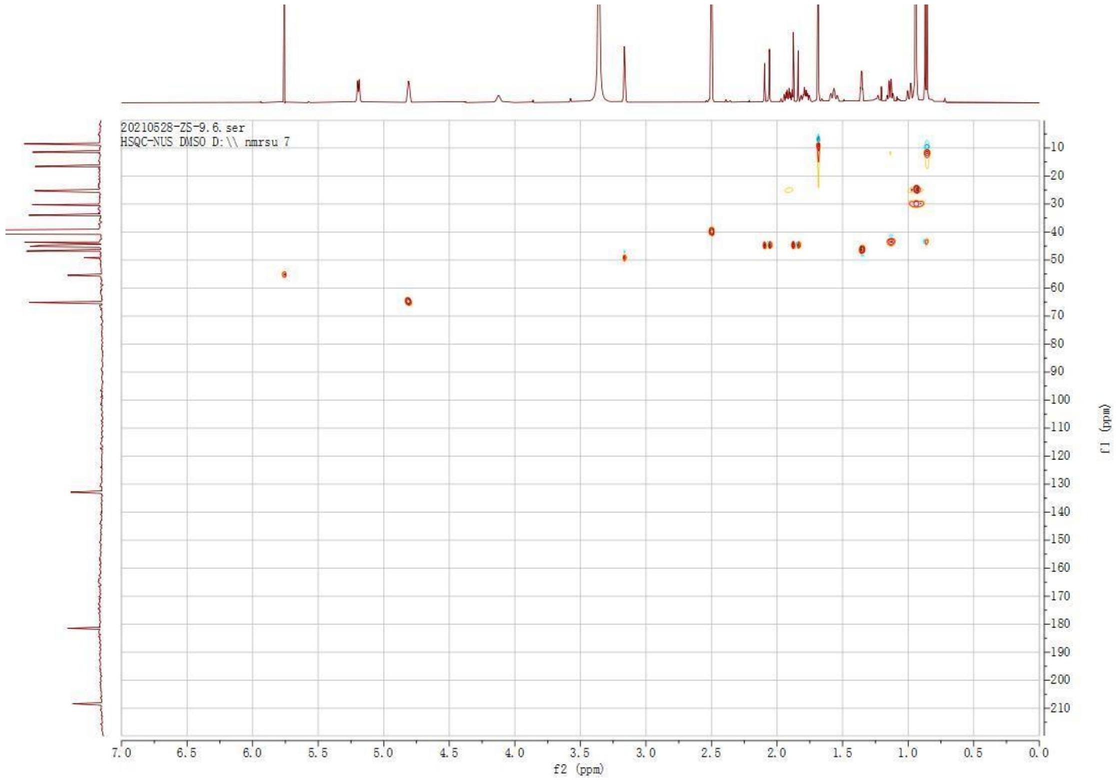

Figure S1 A-9. HSQC spectrum of compound **1**

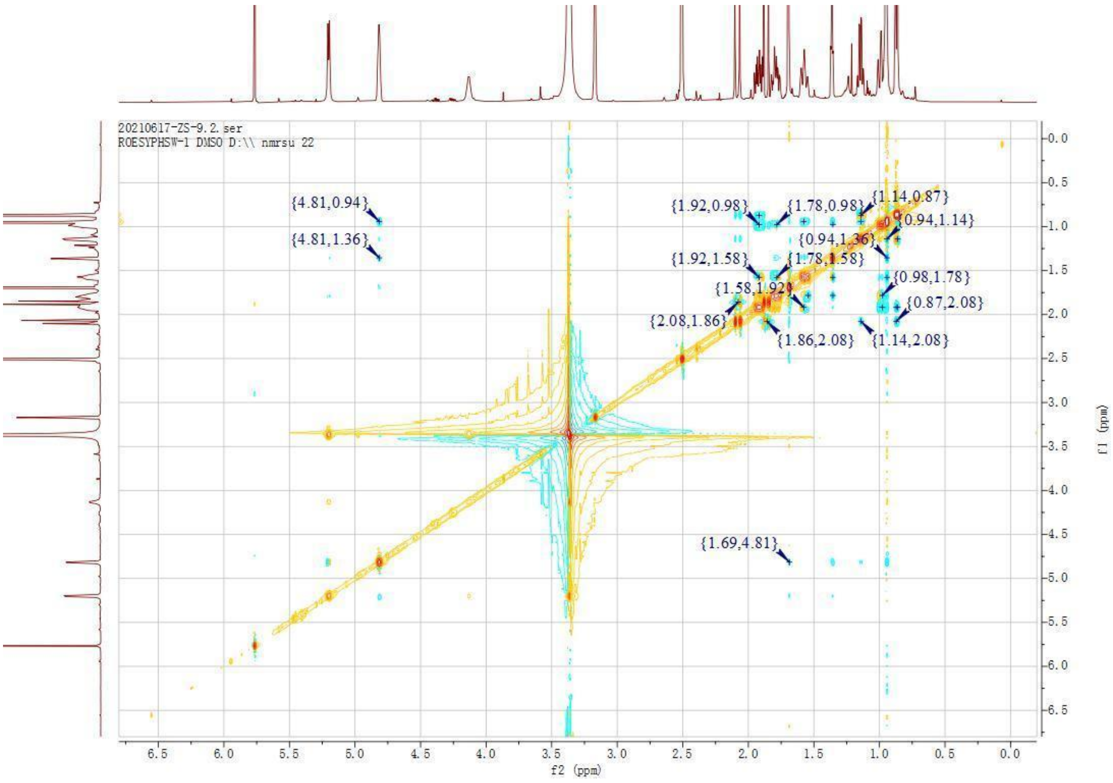

Figure S1 A-10. ROESY spectrum of compound **1**

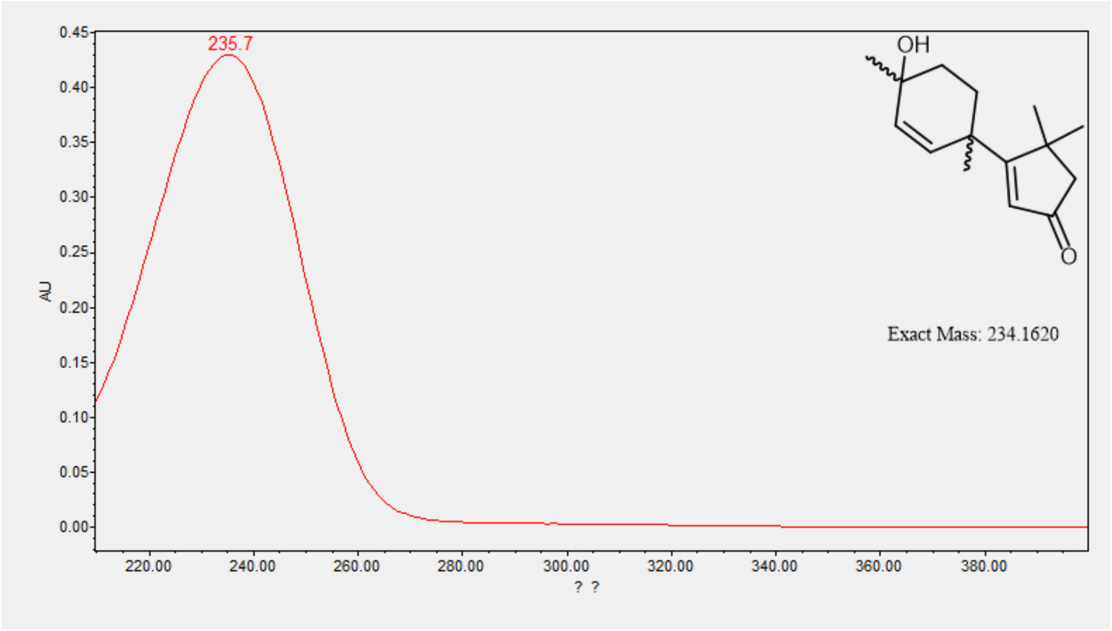

Figure S1 B-1. UV spectrum of compound **2**

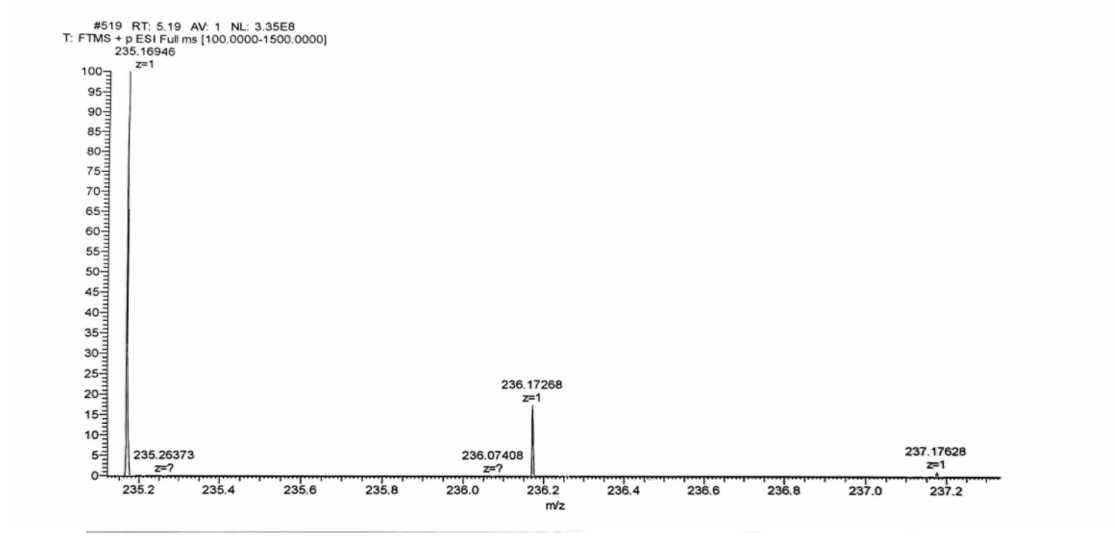

Figure S1 B-2. HRESIMS spectrum of compound **2**

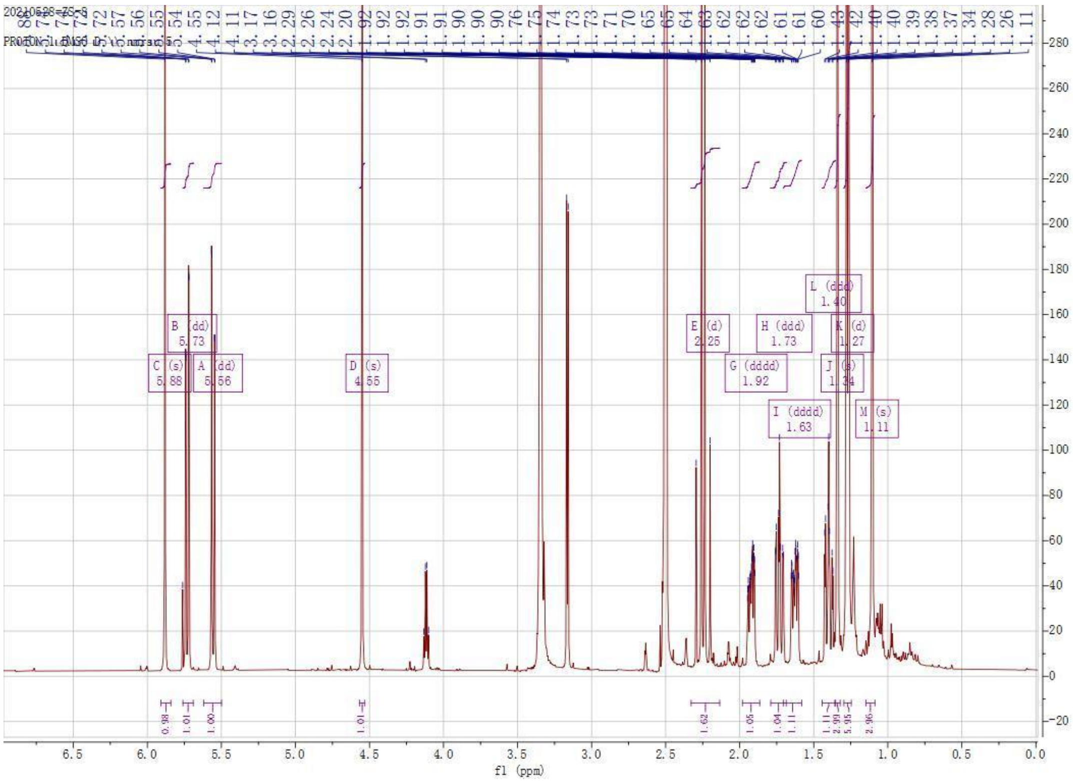

Figure S1 B-3. ^{1}H NMR spectrum of compound **2**

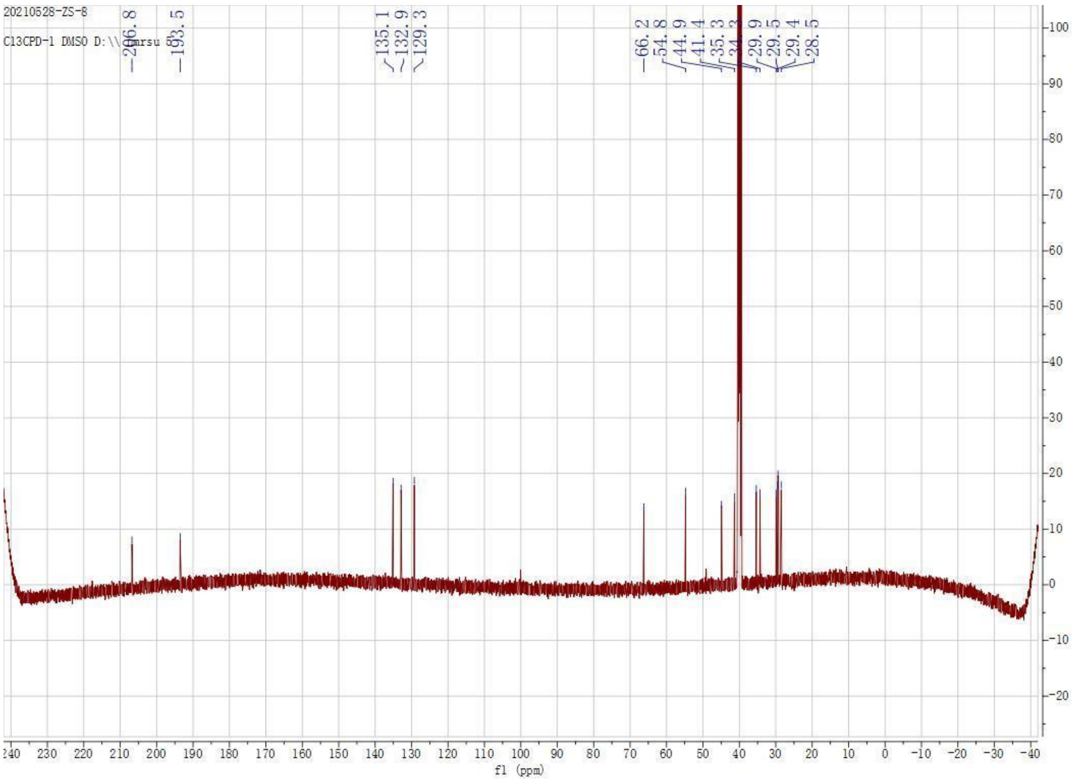

Figure S1 B-4. ^{13}C NMR spectrum of compound **2**

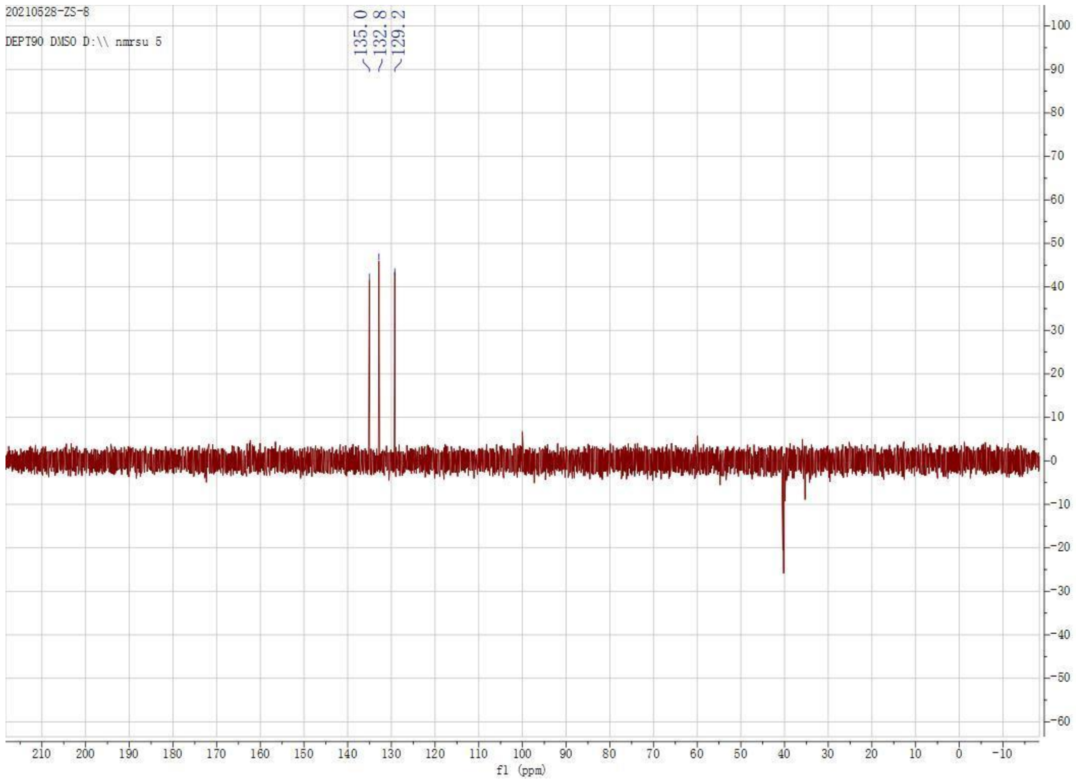

Figure S1 B-5. DEPT-90 spectrum of compound **2**

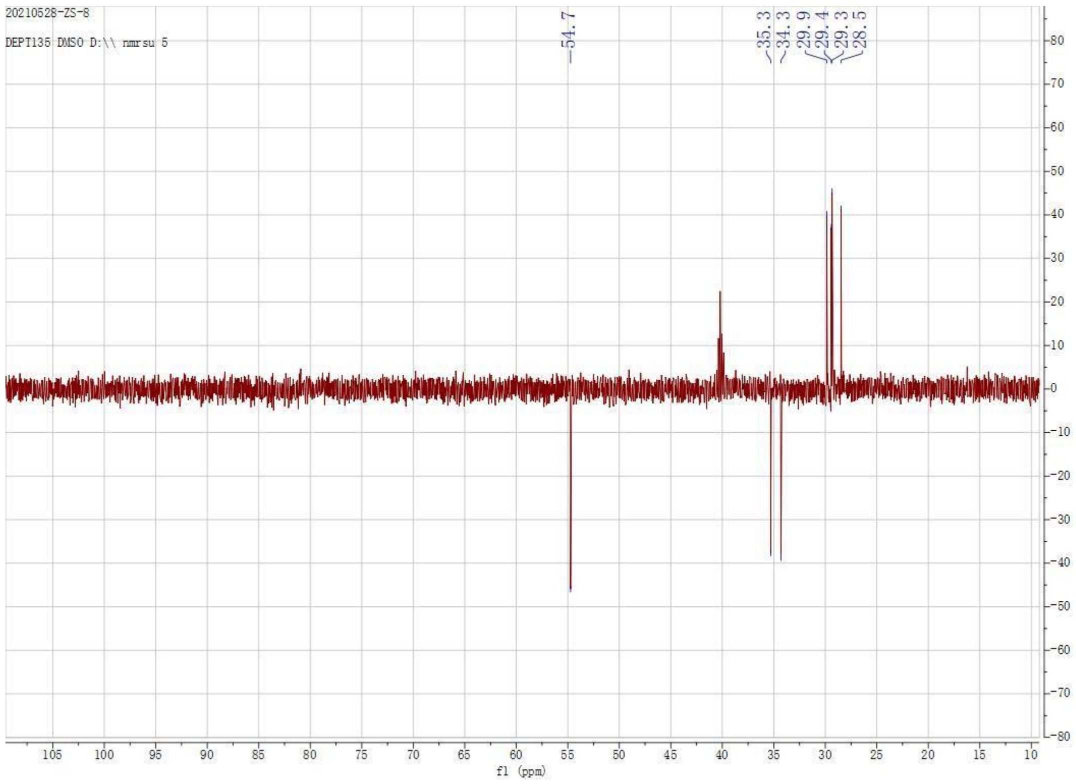

Figure S1 B-6. DEPT-135 spectrum of compound **2**

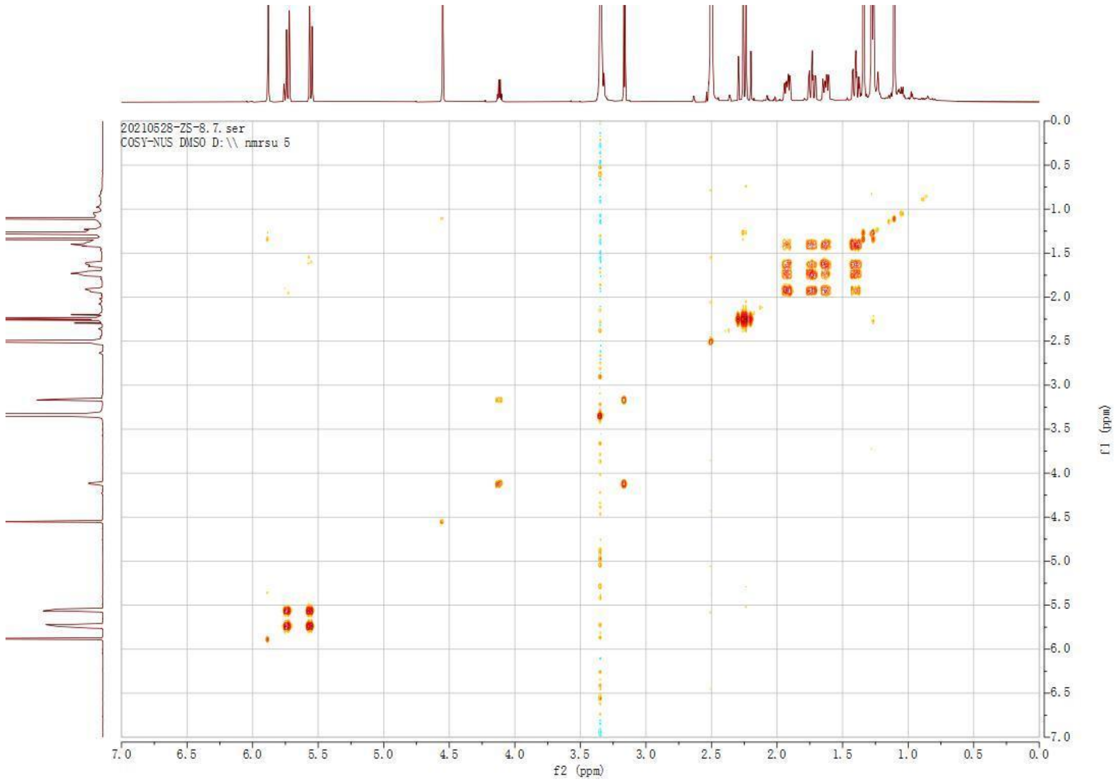

Figure S1 B-9. HSQC spectrum of compound **2**

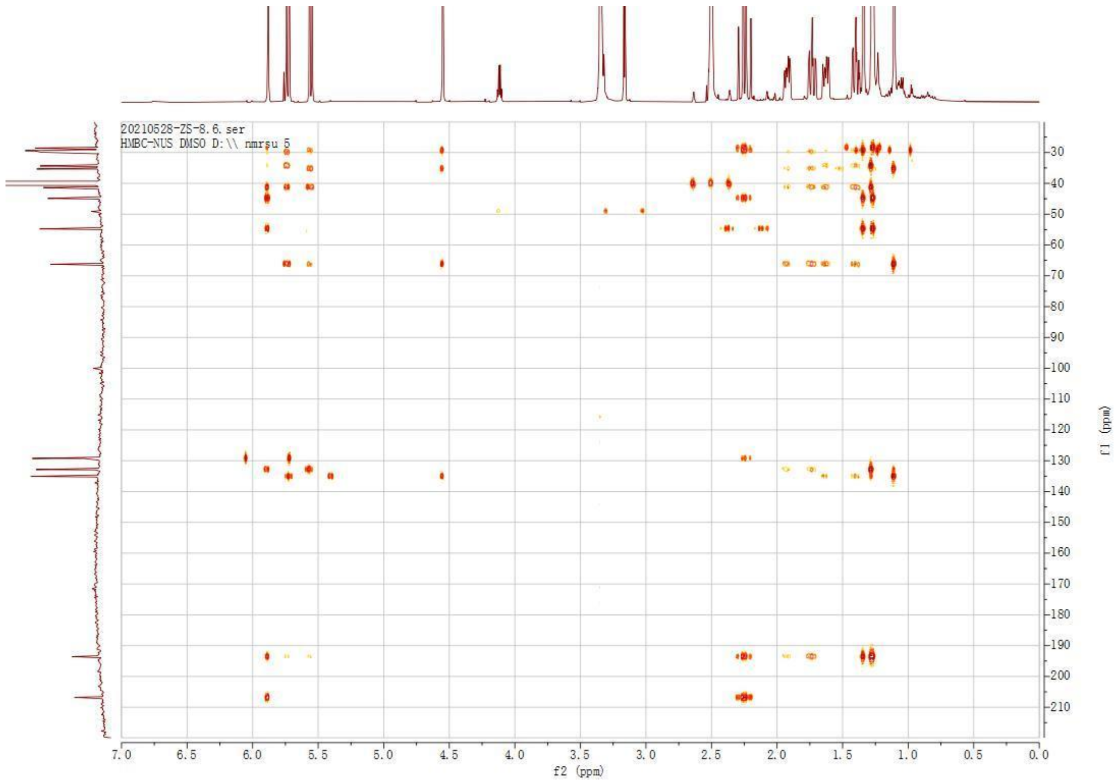

Figure S1 B-10. ROESY spectrum of compound **2**

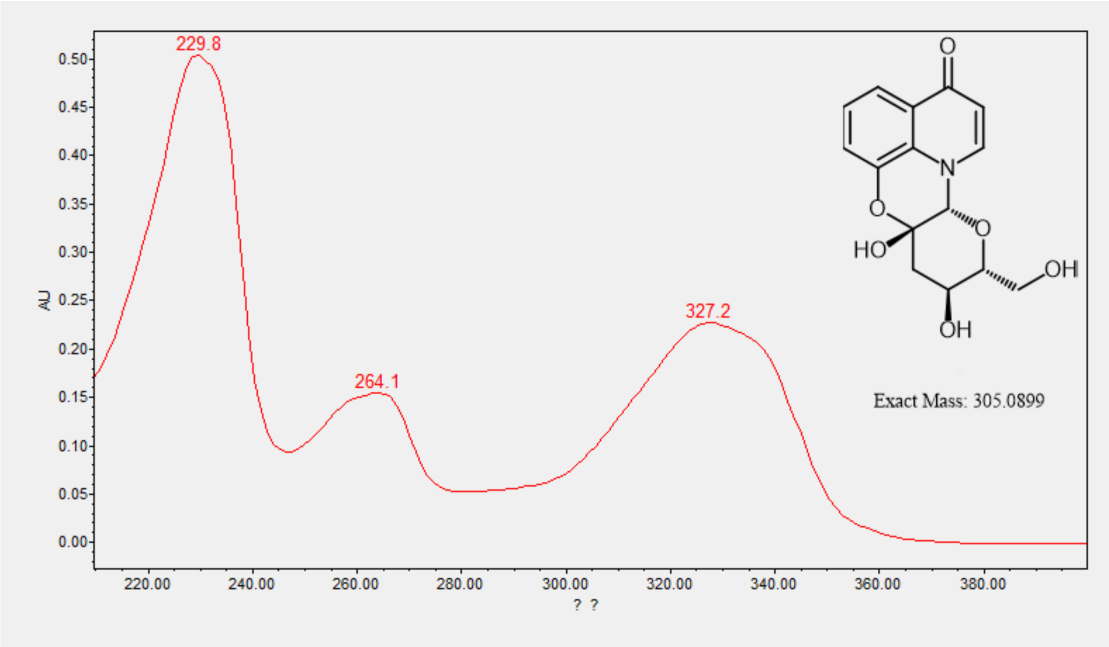

Figure S1 C-1. UV spectrum of compound **3**

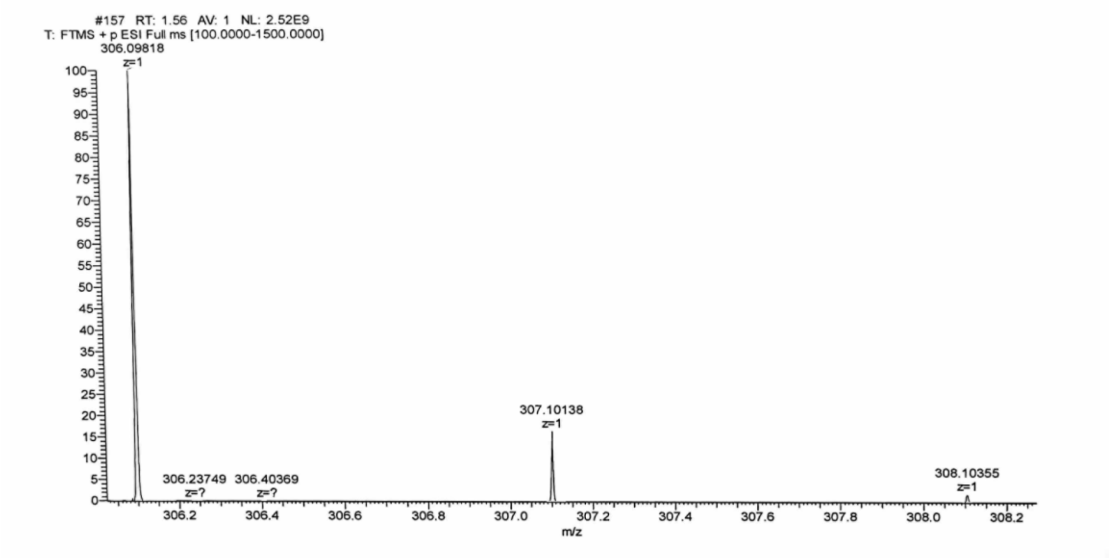

Figure S1 C-2. HRESIMS spectrum of compound **3**

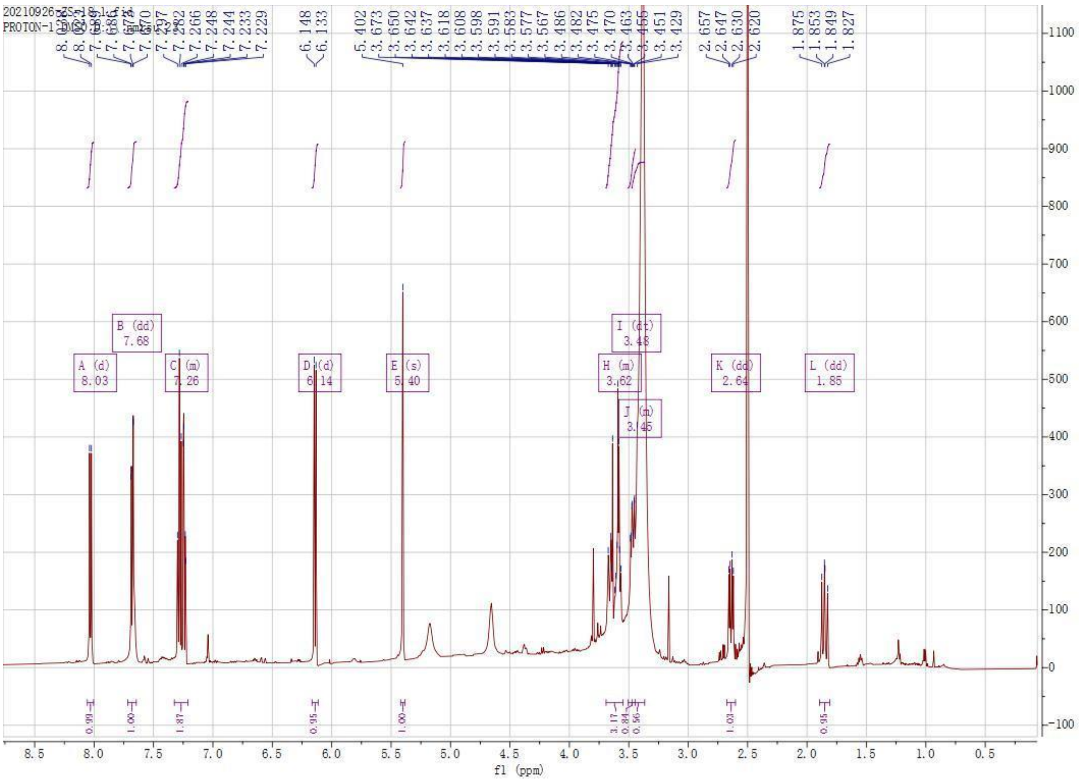

Figure S1 C-3. ^{1}H NMR spectrum of compound **3**

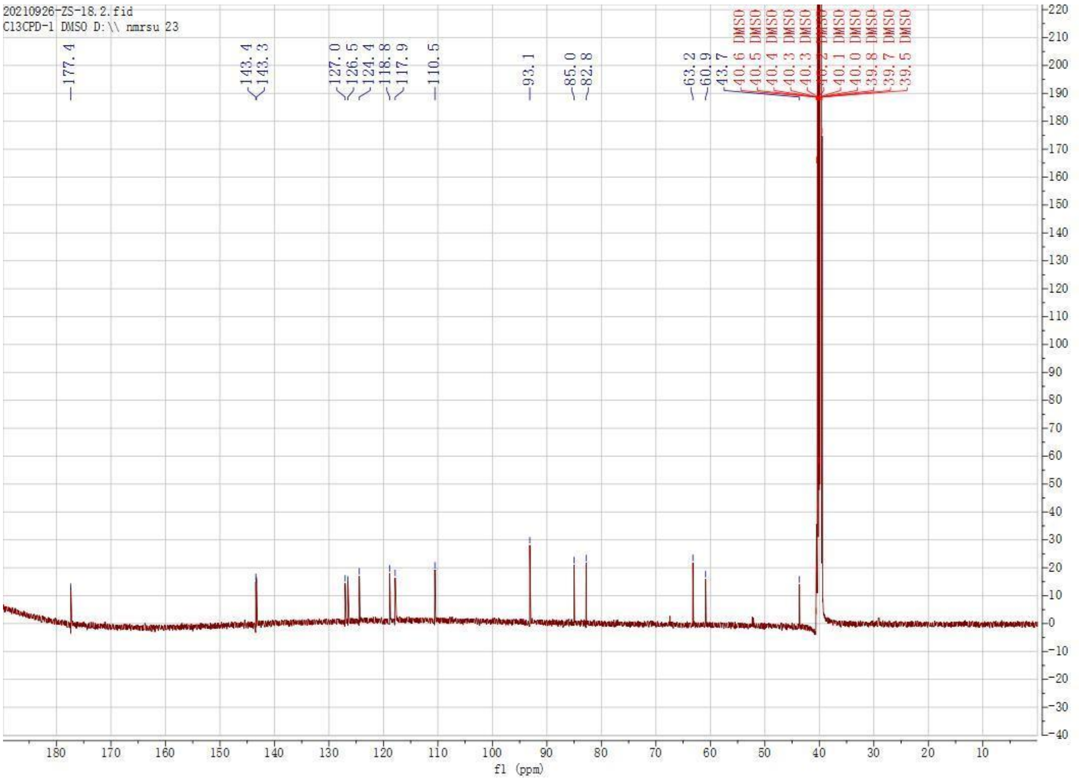

Figure S1 C-4. ^{13}C NMR spectrum of compound **3**

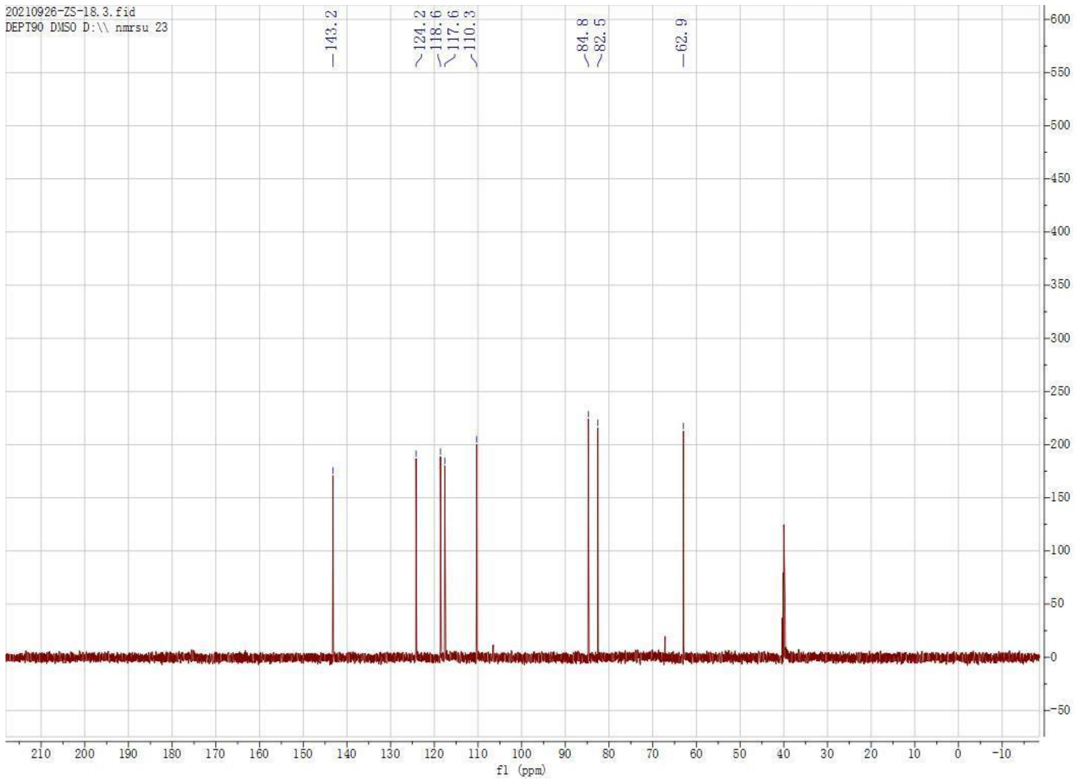

Figure S1 C-5. DEPT-90 spectrum of compound **3**

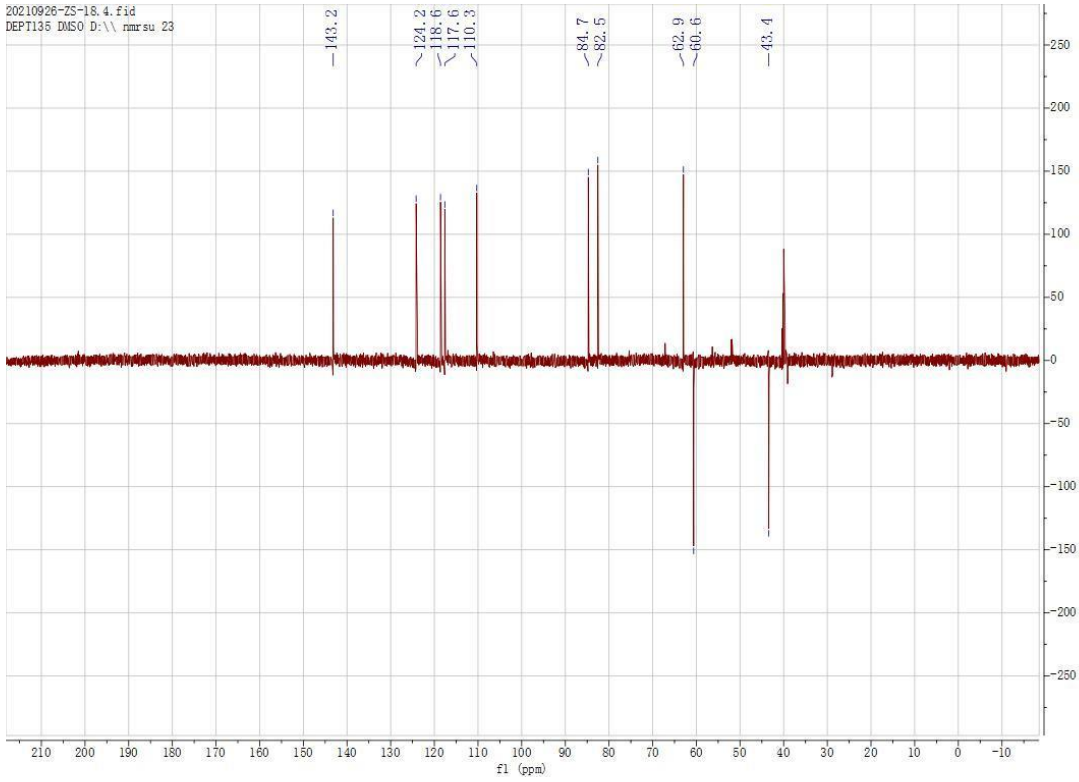

Figure S1 C-6. DEPT-135 spectrum of compound **3**

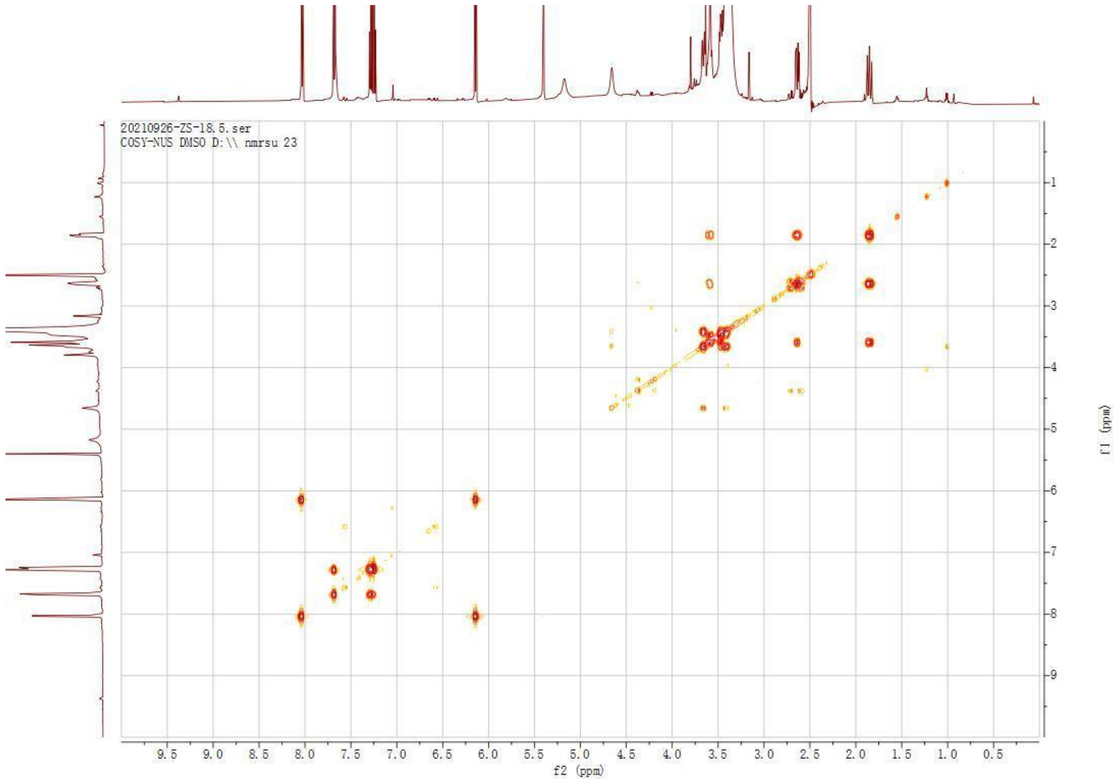

Figure S1 C-7. ^1H-^1H COSY spectrum of compound **3**

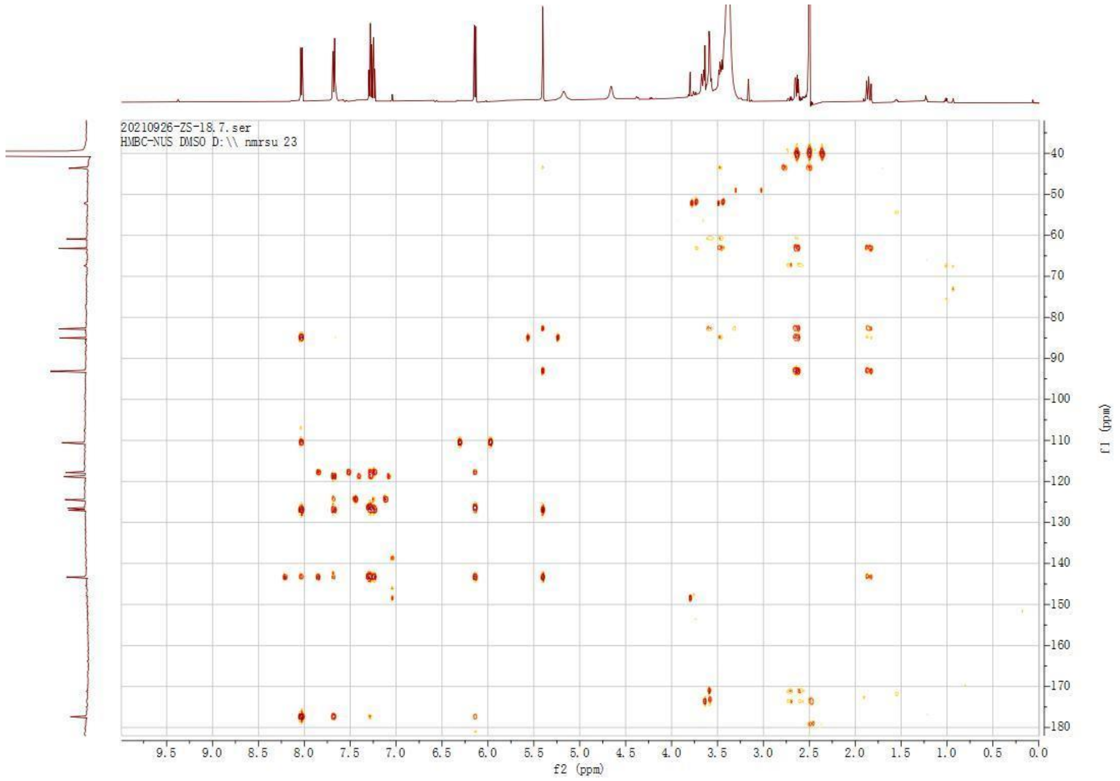

Figure S1 C-8. HMBC spectrum of compound **3**

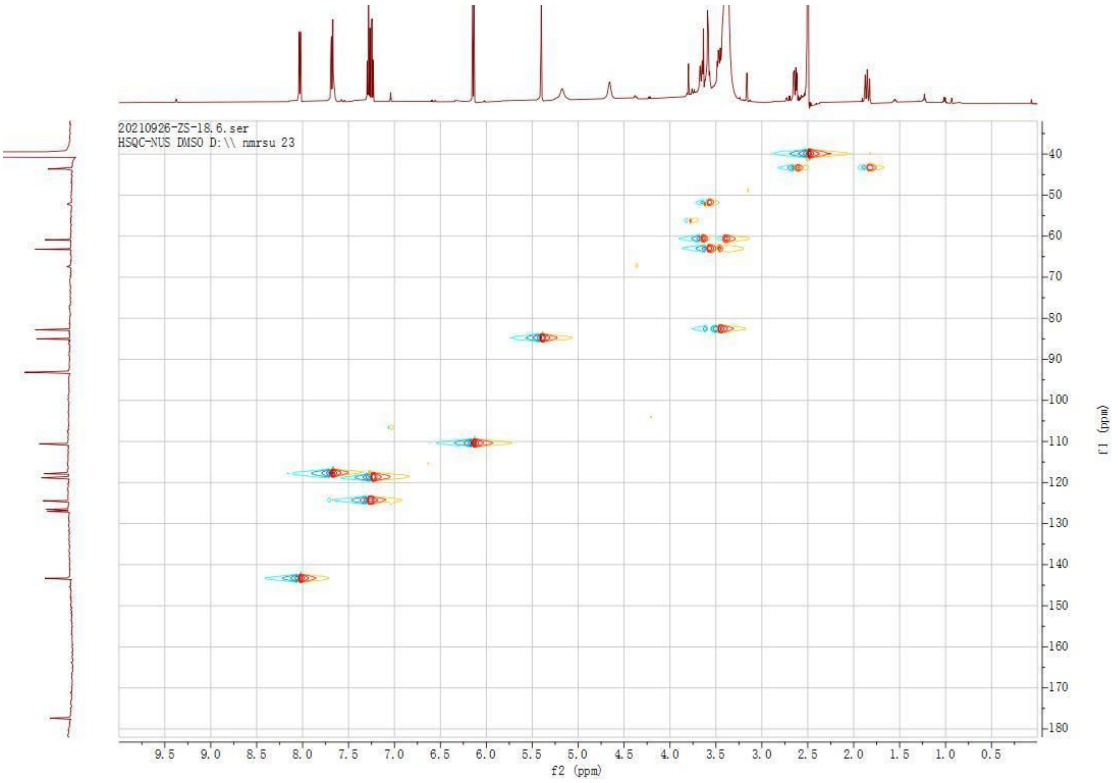

Figure S1 C-9. HSQC spectrum of compound **3**

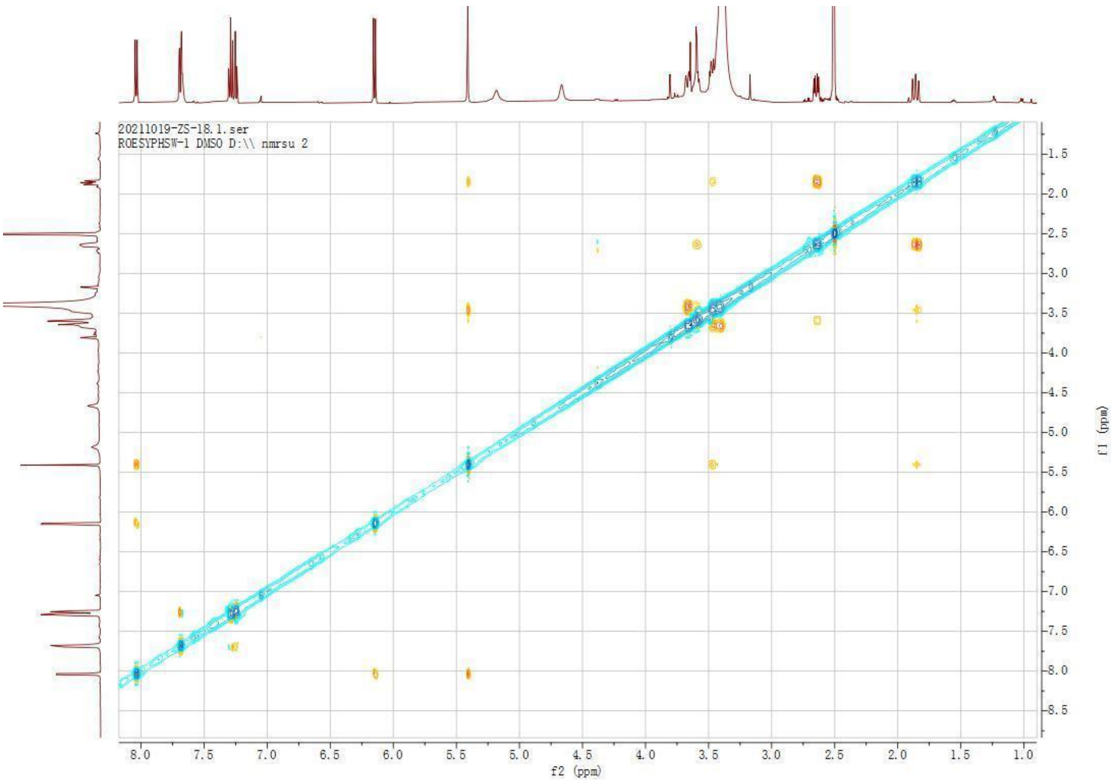

Figure S1 C-10. ROESY spectrum of compound **3**

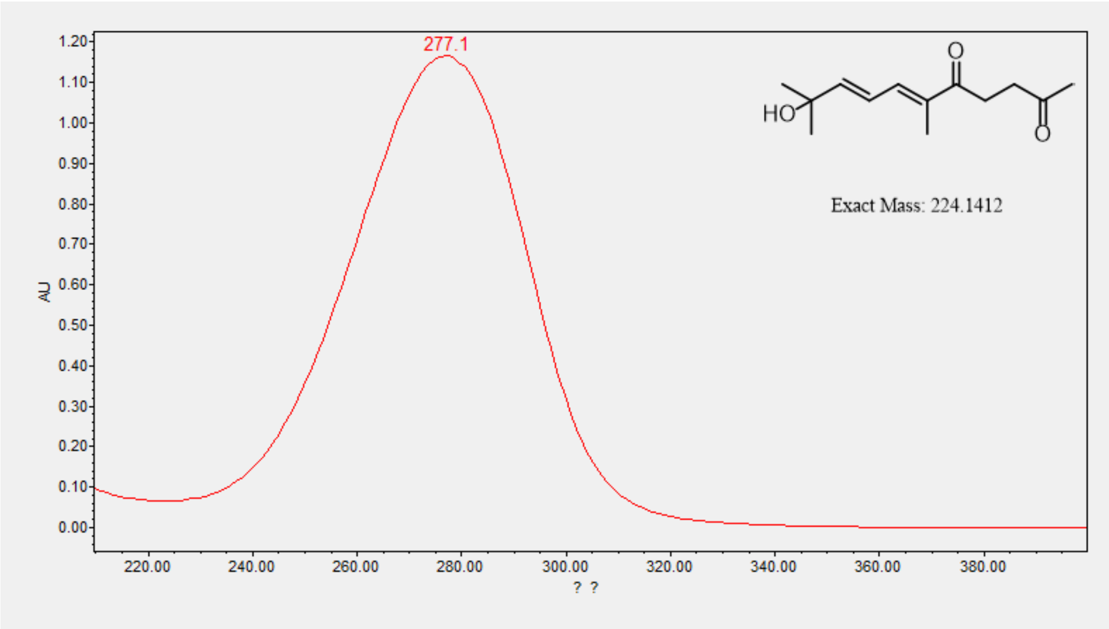

Figure S1 D-1. UV spectrum of compound **4**

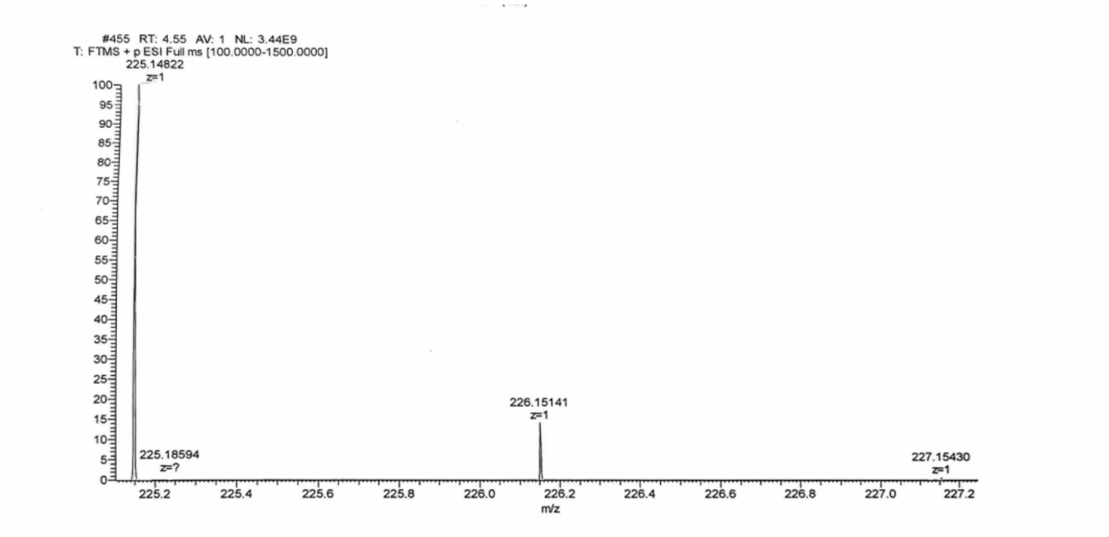

Figure S1 D-2. HRESIMS spectrum of compound **4**

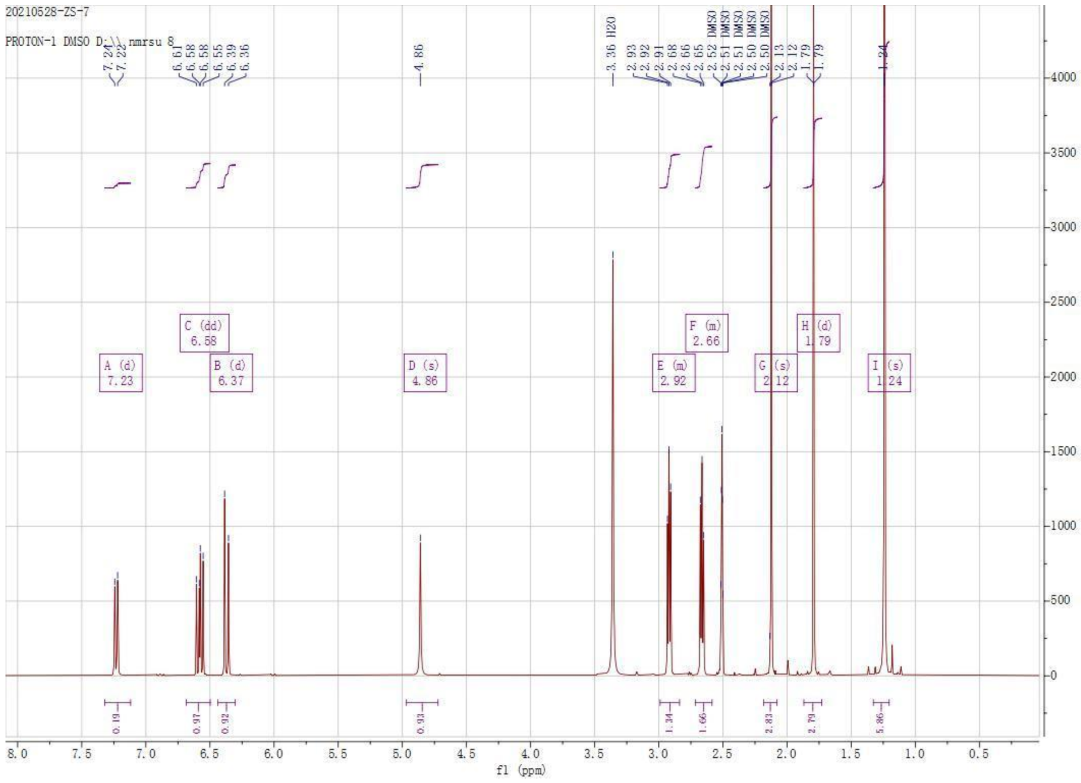

Figure S1 D-3. ^{1}H NMR spectrum of compound **4**

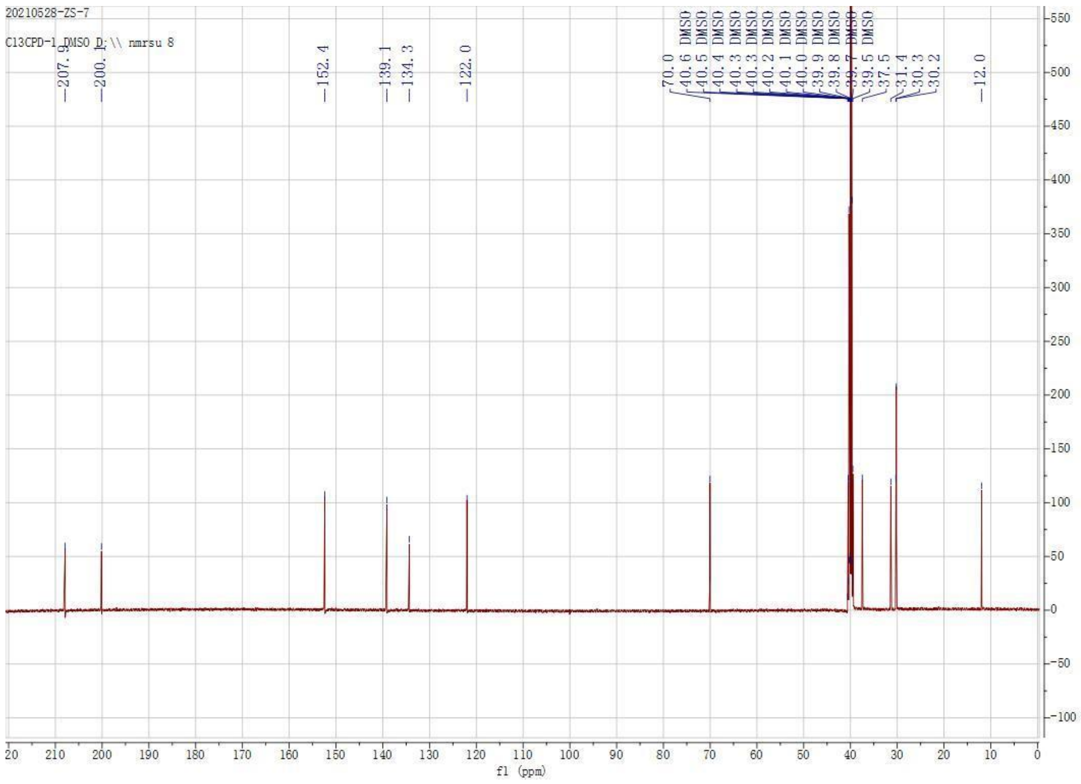

Figure S1 D-4. ^{13}C NMR spectrum of compound **4**

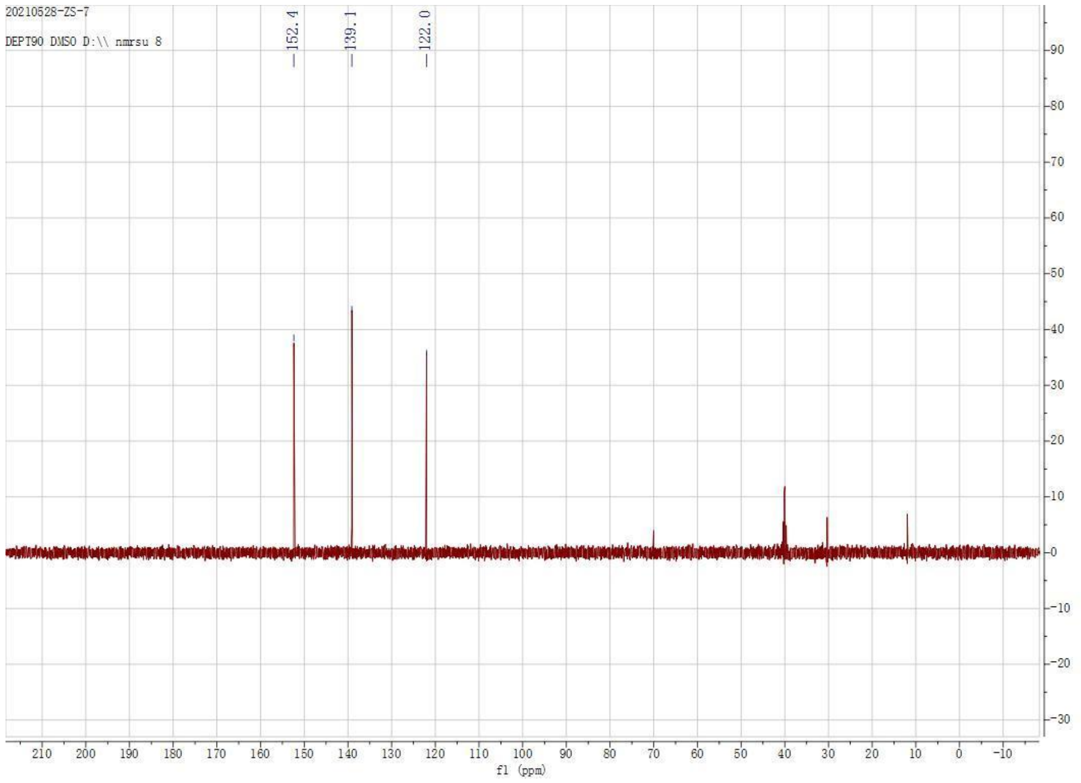

Figure S1 D-5. DEPT-90 spectrum of compound **4**

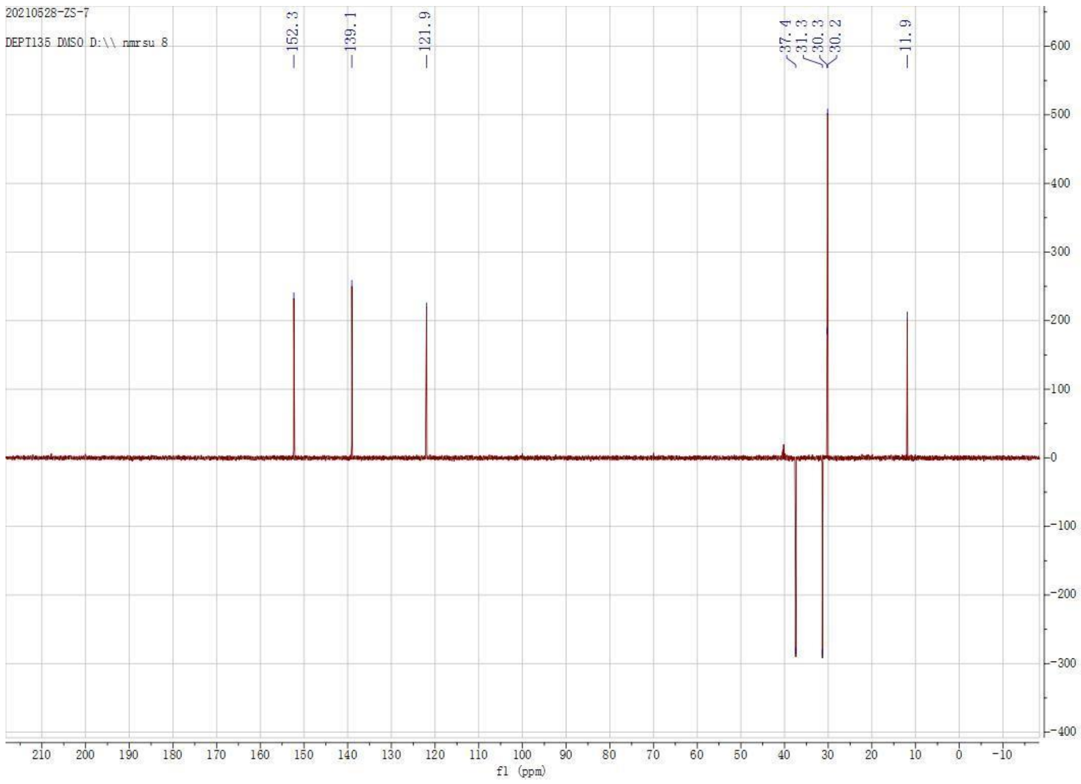

Figure S1 D-6. DEPT-135 spectrum of compound **4**

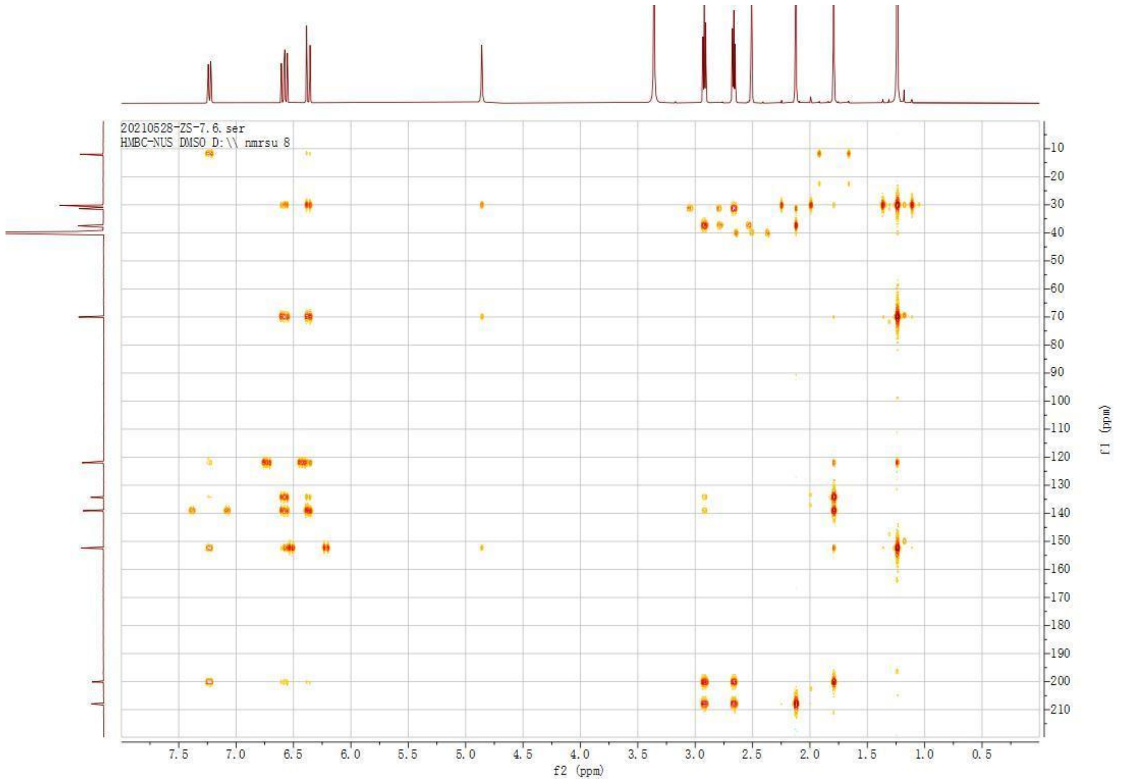

Figure S1 D-7. HMBC spectrum of compound **4**

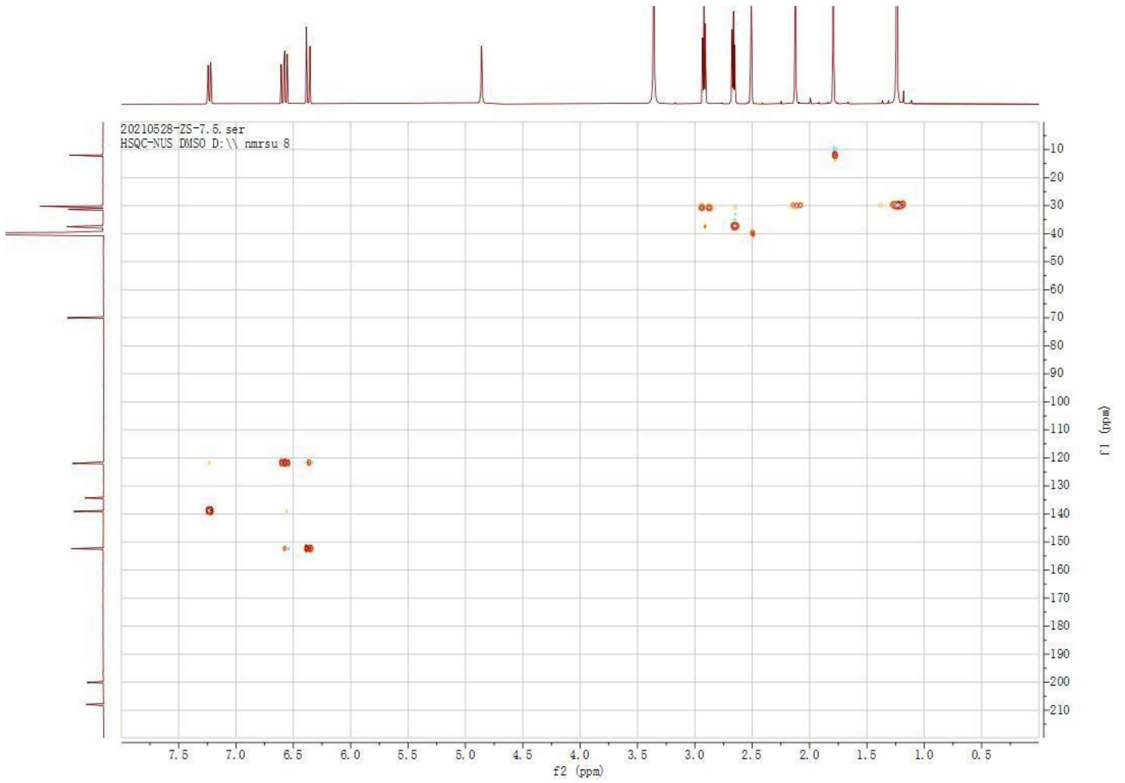

Figure S1 D-8. HSQC spectrum of compound **4**

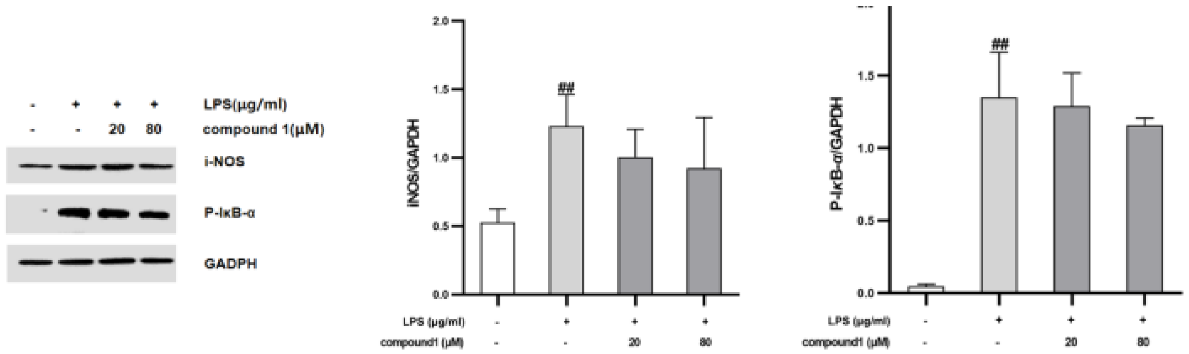

Figure S2 A. Effects of compound **1** on expression of iNOS and *p*-IκB-*α* in lipopolysaccharide (LPS)-simulated BV-2 cells. The results are presented as the mean of triplicates, error bars indicate standard deviations. ## $p < 0.01$ for comparisons with the normal cell group.

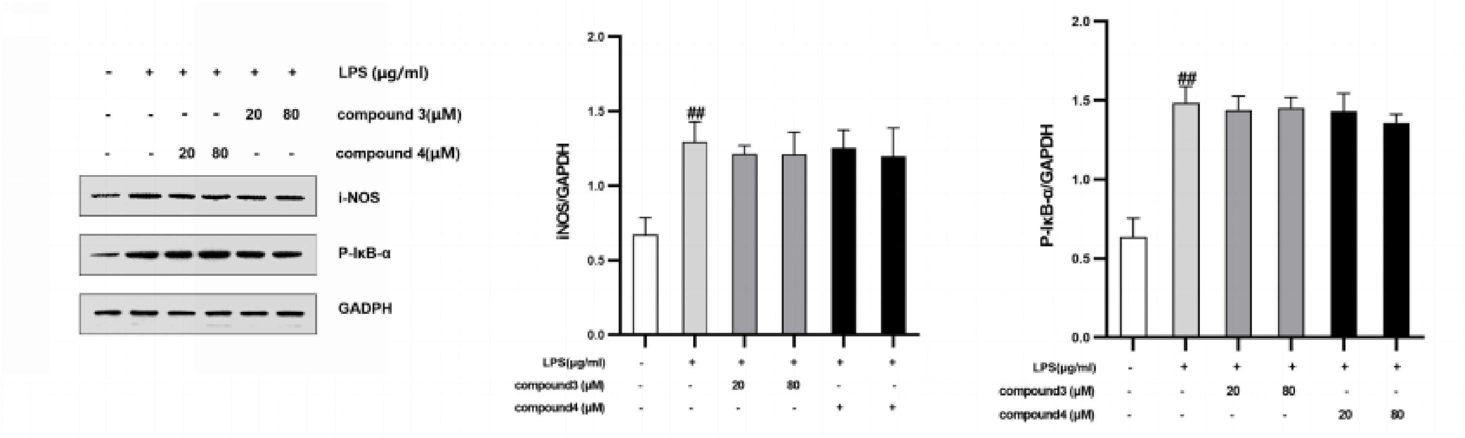

Figure S2 B. Effects of compounds **3** and **4** on expression of iNOS and *p*-IκB-*α* in lipopolysaccharide (LPS)-simulated BV-2 cells. The results are presented as the mean of triplicates, error bars indicate standard deviations. ## $p < 0.01$ for comparisons with the normal cell group.

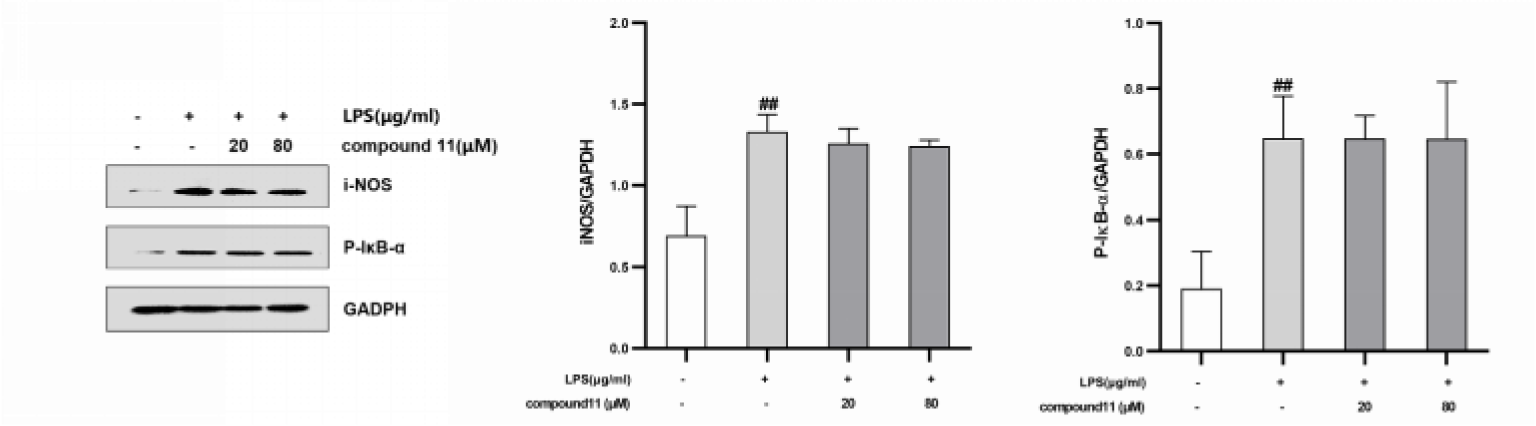

Figure S2 C. Effects of compound **11** on expression of iNOS and *p*-IκB-*α* in lipopolysaccharide (LPS)-simulated BV-2 cells. The results are presented as the mean of triplicates, error bars indicate standard deviations. ## $p < 0.01$ for comparisons with the normal cell group.

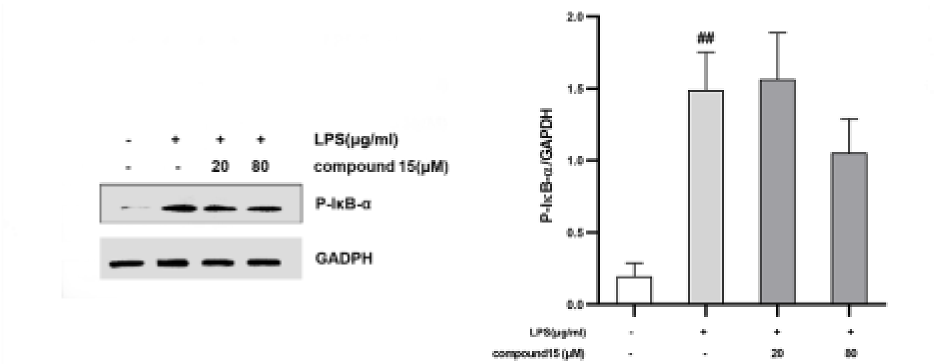

Figure S2 D. Effects of compound **15** on expression of *p*-IκB-*α* in lipopolysaccharide (LPS)-simulated BV-2 cells. The results are presented as the mean of triplicates, error bars indicate standard deviations. ## $p < 0.01$ for comparisons with the normal cell group.

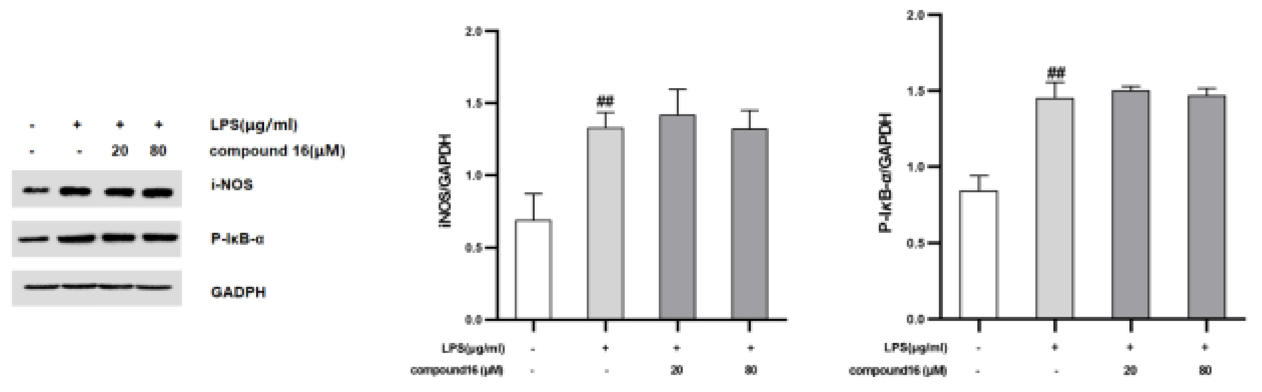

Figure S2 E. Effects of compound **16** on expression of iNOS and *p*-IκB-*α* in lipopolysaccharide (LPS)-simulated BV-2 cells. The results are presented as the mean of triplicates, error bars indicate standard deviations. ## $p < 0.01$ for comparisons with the normal cell group.

Table S1. Main experimental instruments

Experimental procedure	Instrument	Manufacturer
**Column chromatography**	Silica gel (200-300 mesh and 300-400 mesh)	Haiyang, Qingdao, China
	Sephadex LH-20	GE Healthcare, Uppsala, Sweden
**Preparative TLC**	Silica gel plate	Yinlong, Yantai, China
**HPLC and UV**	Waters Acquity H Class UPLC system coupled with a PDA detector 2996	Waters, Milford, MA, USA
	C18 column: YMC-Pack ODS-AQ，250 ×4.6 mm, 5 μm	YMC, Kyoto, Japan
**Preparative HPLC**	Shimadzu LC-6AD instrument with an SPD-20A detector	Shimadzu, Kyoto, Japan
	C18 column: YMC-Pack ODS-AQ，250 × 20 mm, 5 μm	YMC, Kyoto, Japan
**NMR**	Bruker AV-500 spectrometer	Bruker, Zurich, Switzerland
	Solvent: DMSO-*d*6. Internal reference standard: TMS.	Fisher-Scientific, Pittsburgh, PA, USA
**HRESIMS**	Agilent 6540 high-resolution quadrupole time-of-flight mass spectrometer	Agilent, Santa Clara, CA, USA
**CD**	JASCO J-815 CD spectrometer	JASCO, Tokyo, Japan
**XRD**	XtaLAB Synergy R, HyPix diffractometer	Rigaku Americas Corporation, The Woodlands, TX, USA

TLC: thin-layer chromatography; HPLC: high-performance liquid chromatography; UV: ultraviolet spectra; HRESIMS: high resolution electrospray ionization mass spectra; NMR: nuclear magnetic resonance spectra; XRD: X-ray diffraction; CD: circular dichroism spectra; DMSO-*d*6: deuterated dimethyl sulfoxide; TMS: tetramethylsilane.
